# Pain Modulation in Waking and Hypnosis in Women: Event-Related Potentials and Sources of Cortical Activity

**DOI:** 10.1371/journal.pone.0128474

**Published:** 2015-06-01

**Authors:** Vilfredo De Pascalis, Vincenzo Varriale, Immacolata Cacace

**Affiliations:** Department of Psychology, Sapienza University of Rome, Rome, Italy; Université catholique de Louvain, BELGIUM

## Abstract

Using a strict subject selection procedure, we tested in High and Low Hypnotizable subjects (HHs and LHs) whether treatments of hypoalgesia and hyperalgesia, as compared to a relaxation-control, differentially affected subjective pain ratings and somatosensory event-related potentials (SERPs) during painful electric stimulation. Treatments were administered in waking and hypnosis conditions. LHs showed little differentiation in pain and distress ratings between hypoalgesia and hyperalgesia treatments, whereas HHs showed a greater spread in the instructed direction. HHs had larger prefrontal N140 and P200 waves of the SERPs during hypnotic hyperalgesia as compared to relaxation-control treatment. Importantly, HHs showed significant smaller frontocentral N140 and frontotemporal P200 waves during hypnotic hypoalgesia. LHs did not show significant differences for these SERP waves among treatments in both waking and hypnosis conditions. Source localization (sLORETA) method revealed significant activations of the bilateral primary somatosensory (BA3), middle frontal gyrus (BA6) and anterior cingulate cortices (BA24). Activity of these contralateral regions significantly correlated with subjective numerical pain scores for control treatment in waking condition. Moreover, multivariate regression analyses distinguished the contralateral BA3 as the only region reflecting a stable pattern of pain coding changes across all treatments in waking and hypnosis conditions. More direct testing showed that hypnosis reduced the strength of the association of pain modulation and brain activity changes at BA3. sLORETA in HHs revealed, for the N140 wave, that during hypnotic hyperalgesia, there was an increased activity within medial, supramarginal and superior frontal gyri, and cingulated gyrus (BA32), while for the P200 wave, activity was increased in the superior (BA22), middle (BA37), inferior temporal (BA19) gyri and superior parietal lobule (BA7). Hypnotic hypoalgesia in HHs, for N140 wave, showed reduced activity within medial and superior frontal gyri (BA9,8), paraippocampal gyrus (BA34), and postcentral gyrus (BA1), while for the P200, activity was reduced within middle and superior frontal gyri (BA9 and BA10), anterior cingulate (BA33), cuneus (BA19) and sub-lobar insula (BA13). These findings demonstrate that hypnotic suggestions can exert a top-down modulatory effect on attention/preconscious brain processes involved in pain perception.

## Introduction

Human pain is a multi-faceted protective experience involving the activity of sensory-discriminative, affective-emotional, attention-cognitive and behavioral systems [1–6]. Melzack and Casey [7] proposed that this experience reflects the mutual interaction of sensory, affective and cognitive dimensions and, thus, requires the integrated activity of a widely distributed network of neurons, extending throughout widespread areas of the brain (a neuromatrix), necessary to generate the neurosignature pattern for pain. This view implied that pain is the product of the activity of a multidimensional and distributed neural network rather than of a “brain pain centre” usually triggered by sensory inputs. This network, often referred to as the “pain matrix” (PM), is viewed as representing the activity induced by the intensity and unpleasantness of a nociceptive stimulus. However, recent studies [8] have beset the very concept of a specific pain-related network, claiming that most regions present in the PM are part of a basic nonspecific salience-detection system, activated by the occurrence of potentials threats detected for the body’s integrity, regardless of the sensory channel through which these events are conveyed [9,10]. This view has been provided suggesting that the brain responses to nociceptive stimuli, as measured using functional neuroimaging techniques (i.e., EEG, MEG, fMRI, PET), do not reflect nociceptive-specific brain activities, but, instead, brain activities equally involved in processing nociceptive and non-nociceptive salient sensory inputs. Therefore, it has been suggested that the term “pain matrix” should be used with caution, because it misleadingly implies that the recorded responses are specific for pain [9]. However, very recently, PM has been reconceptualized as a fluid system composed of several interacting networks [11]. A first-order nociceptive matrix responsible for the earliest responses to noxious stimuli (i.e., spinothalamic sensory cortices, brainstem, bilateral thalamus, posterior insula, medial parietal operculum and mid-cingulate cortex) ensures the bodily specificity of pain and is the only one whose destruction entails selective pain deficits [12]. The transition from cortical nociception to multiple attentional-affective and cognitive modulations, necessary for conscious perception of pain, requires the recruitment of a second order-network of not nociceptive-specific cortical regions including anterior cingulate cortex (ACC) [13–15], premotor cortex [16], dorsolateral prefrontal cortex (DLPFC) [17], posterior parietal, prefrontal and anterior insular areas, and cortical representations of non-painful tactile stimuli, highly aligned with nociceptive maps [18–20]. Since pain experience can be modified as a function of beliefs [21], expectations [22] and placebo [23], this new reconceptualization has suggested that this is done through the activity of third-order areas, including the orbitofrontal and perigenual/limbic networks. In this view, the neural substrate of the pain experience is conceived at progressively different levels of higher-order cortical networks, from cortical nociception to conscious experience. The role of different regions being dependent on the context in which the stimuli are delivered, has been put forward by a few investigators (eg, [24, 25]) and conscious experience called "pain" is subjected to reappraisal by internal states, feelings and beliefs prior to stabilization into memory stores.

Using a variety of stimulation methods, painful events have been shown to produce an early contralateral response in the primary somatosensory cortex (S1) reflecting the sensory-discriminative aspect of pain [26–29], followed by a more distributed contralateral activity in the 120–160 ms range (N1 wave) that is localized in the parietal operculum (S2) [30–32] and is found to be modulated by a top-down attentional control [2]. A late P2 response (200–300 ms) [33], bilaterally distributed, is known to be modulated by both bottom-up sensory discriminative attentional effect and top-down cognitive or affective evaluation of nociceptive stimulations [2, 34–39]. This wave increases as a function of both stimulus intensity and reported pain [40] and P2 amplitude has been proved to increase with the novelty of nociceptive stimuli, a finding that parallels the novelty-P3a modulation effect observed for auditory, visual, and tactile event-related potentials (ERPs) [41]. A likely source of the P2 is the ACC [42,43], which is sensitive to pain modulation in hypnosis [43,44]. Hypnotic analgesia is associated with changes in pain thresholds and pain correlates with brain activity [45–47] and somatosensory event-related potentials (SERPs) [47–49]. High hypnotizable individuals (HHs) generally display larger reductions in perceived pain, and smaller N140 and P200 SERP amplitudes to painful stimuli when compared to low hypnotizable individuals (LHs) [48, 50]. Research has evidenced that obstructive hallucination of noxious stimulation in hypnosis reduces pain sensation and the P250/P300 amplitude of the SERPs [48,50,51], while this wave is enhanced by hypnotic hyperalgesia [49,52]. Mainly previous studies [49,52] have shown that the locus of hypnotic influence does not lie in the initial sensory experience itself, but rather in the cognitive-emotional component of the information processing, although the hypothesis that hypnosis influence may also affect initial sensory experience cannot be ruled out [36]. Brain imaging studies have proved that hypnotic analgesia may produce activity changes in a number of brain regions, including the mid and anterior cingulate cortex, insula, perigenual cortex, pre-supplementary motor cortex, brainstem, and thalamus [45,46,53,54,55]. Hypnotic suggestions to increase or decrease pain intensity have been associated with significant changes in pain-related activity within S1 [47], in contrast to previous studies [56,57] in which specific modulation of pain unpleasantness, independently of pain intensity, produced pain-related changes within the ACC. Research has suggested that the early N1 wave (120–160 ms) of the SERP may represent an index of early stage of sensory processing related to the ascending nociceptive input, whereas the later P2 wave (200–300 ms) may reflect a later stage of processing related, directly or indirectly, to the perceptual outcome of this nociceptive input [58].

In the EEG based search for the acknowledged multiple brain generators involved in pain perception, the superiority of standardized low-resolution brain electromagnetic tomography (sLORETA) [59,60] over the other linear inverse solution methods has been demonstrated [61]. Nir and collaborators [17], using sLORETA method, identified well-known pain-related regions induced by thermal stimulation, as the bilateral primary somatosensory (S1) and anterior cingulate cortices, the contralateral operculoinsular (OI) and dorsolateral prefrontal (DLPFC) cortices. These authors, using a multivariate regression analysis, were able to distinguish the contralateral S1 as the only region whose activation magnitude significantly predicted the subjective perception of noxious stimuli. On this basis, aim of the present study was to extend Nir et al's findings to pain induced by electric pulse stimulation. We expected that activity in the S1 region, contralateral to the stimulated side, should be associated with subjective pain rating scores in a waking baseline condition. We also want (a) to examine how pain modulation induced by experimental manipulations in waking and hypnosis relates to brain activity and (b) to highlight brain responses coding for pain/distress and (c) if the brain activity of pain/distress coding may change depending on the experimental treatment and condition. Moreover, aims of the present study were to assess the relation between the effects of experimental manipulations on N100 and P200- SERP components, their sLORETA-based activations and the magnitude of the changes reported in subjective pain perception. In particular, we want to validate our previous pain-hypnosis SERP findings, i.e., that hypnotic analgesia with HHs can reduce both N140 and P200 wave [48], and those reported by Ray and collaborators [49], i.e., that hypnotic suggestion with HHs modulates the later component, but not the earlier one, of the SERPs. We also expected that (1) HH and LH individuals, during a control-baseline treatment in waking and hypnosis conditions, show small self-report or SERP differences in response to noxious stimuli, and that (2) HHs during hypnosis report differential pain and distress experiences and SERP changes depending on the direction of hypnotic suggestions (i.e., to enhance or reduce pain perception).

A secondary aim of the present study was to identify the major cortical regions sensitive to individual differences in hypnotizability and hypoalgesia and hyperalgesia treatments by using sLORETA source localization [59,60] of N140 and P200 waves. According to the recent PM view [11], we expected that hypoalgesia and hyperalgesia manipulations, during hypnosis with HHs, should modulate activity within a number of second- and third-order not nociceptive-specific regions of PM including somatosensory cortex (highly aligned with nociceptive maps [18–20]), anterior cingulate cortex (ACC), premotor cortex, posterior parietal, insular areas and orbitofrontal/limbic networks.

## Methods

### Ethics statement

Participant upon arrival to the EEG lab were first informed about the use of hypnosis, and all of them were informed that, during EEG recording, they would receive painful electric stimulations in waking and hypnosis condition. All of them gave their written informed consent for participation.

The research was conducted according to the ethical standards of the American Psychological Association (APA), and according to the principles expressed in the Declaration of Helsinki. The study was approved by the Ethics Committee of the Department of Psychology, La Sapienza University of Rome, Italy (2008).

### Subjects

From a group of 79 right-handed women (aged 20–34, M = 25.1, SD = 3.5) naïve volunteers, 10 high hypnotizable and 10 low hypnotizable subjects were recruited through university courses by advertisements. Handedness was measured by the Italian version of the Edinburgh Inventory Questionnaire [61]. Subjects were selected by first using the Harvard Group Scale of Hypnotic Susceptibility, Form A (HGSHS:A) [62, 63] and, about a week later, the Stanford Hypnotic Susceptibility Scale, Form C (SHSS:C) [64,65]. Subjects were considered to be highly hypnotizable subjects (HHs) when their scores on both the HGSHS:A and SHSS:C (M = 1.1, SD =. 4, and M = 9.7, SD =. 6, for the HGSHS:A and SHSS:C, respectively) were one standard deviation above the group mean of a larger sample tested in our laboratory (N = 79, SHSS:C M = 6.4, SD = 3.0; HGSHS:A M = 6.5, SD = 2.2); an equivalent but opposite deviation designated the low hypnotizable subjects (LHs: M = 1.6, SD =. 6, and M = 2.9, SD = 1.5, respectively for the HGSHS:A and SHSS:C). The assessment of hypnotizability was carried out by two different trained women operators about two weeks prior to EEG recording session. During this session, hypnosis was induced for the third time using an Italian translation of the original American protocol of the Stanford Hypnotic Clinical Scale (SHCS) [66]. Only physically healthy participants were included. Inclusion criterion demanded the absence of any lifetime history of significant psychiatric or neurologic disease, drug abuse, head trauma or loss of consciousness, treatment with antipsychotic medication, substance abuse or dependence use of amphetamine or cocaine (excluding caffeine and nicotine) and the absence of medical conditions that might interfere with pain sensitivity (e.g., high blood pressure, diabetes mellitus, asthma, heart diseases, frostbite, arthritis, Raynaud’s syndrome, post-trauma to hands). This information was obtained using a self-report questionnaire. The subjects were asked to refrain from smoking or drinking coffee for at least three hours before the EEG recording. Care was taken to ensure that participants had no information about their level of hypnotic ability and of the relevance of hypnotic ability in pain modulation. Among selected participants, 10 were unhabitual smokers since they smoked no more than 15 cigarettes per day. The subjects were all women since there are reports indicating that women are significantly more susceptible to hypnosis than men [67, 68], although more recent research has demonstrated that gender difference seems to be rather small even when found [69] and that males and females process the same environmental event with different patterns of EEG hemispheric asymmetry [70,71]. Participants who were in their menstrual period were invited for electrophysiological recordings on another occasion between the 5th and 11th day after the onset of menses.

### Stimuli and procedure

All participants went through three experimental sessions, 1 week apart. These sessions were identical except that EEG was recorded only in the third session and is the basis of this present report. The purpose of the first session was to administer the HGSHS:A to naïve participants in groups of 15–20, and to make participants familiar with the experimental procedures. In the second session the SHSS:C was administered singularly to each participant. Selected subjects for hypnotic susceptibility (10 HHs and 10 LHs) participated in the third session wherein painful stimuli were delivered. Stimuli were unipolar electric shocks (2 ms) applied to the palmar surfaces of the distal and medial phalanges of the middle finger of the right hand. A constant current stimulator (Digimiter, Mod DS7A) served to generate these stimuli. Two silver-silver-chloride cup electrodes (1 cm in diameter) were filled with an electro-conductive hypo allergic cream and impedance was kept below 30 kΩ. For each participant we determined first sensory threshold and then pain threshold just before of EEG recordings. Throughout the experiment, electric pulses were applied with a frequency of 1.150 Hz and duration of 2 ms. Pain thresholds were determined for the right middle finger using the method of limits. This stimulation approach was derived from a previous study reported by Ray and collaborators [49], since one of the aims of the present study was to replicate previous SERP wave findings reported by these authors, in relation to hypnotic susceptibility and suggestion. Using this method, the experimenter, starting from an intensity of. 05 mA, increased, then decreased, the current slowly by steps of. 05 mA. The subjects were asked first to indicate when they first experienced the electric pulse and second when they judged the sensation as slightly painful. The stimulus intensity was then further increased to 50% over the intensity that was felt as painful. The electric shock intensity then was continuously decreased, and the subjects were asked to indicate when the stimuli were no longer painful and when they didn’t feel the pulse any longer. This procedure was repeated two times. For the threshold calculations only data from the final two series were used. Stimulus intensities used during the EEG recordings were 50% higher than the individual pain thresholds. The current intensity values of pain thresholds obtained for high and low hypnotizable subjects were of 4.36±0.98 mA and 4.09±0.91 mA, respectively.

Following threshold determinations, the participant sat in a comfortable armchair in a small, darkened, noise-reduced box. During both waking and hypnosis conditions, three treatments for pain modulation were given: (1) hyperalgesia suggestion, aimed at increasing the perception of a painful stimulation; (2) hypoalgesia suggestion, devoted to reduce the perception of painful stimulation; (3) a relaxation-control suggestion, aimed at producing a simple muscle relaxation. A baseline eyes-closed resting period of 1.5 min was given before the administration of each pain treatment. For hyperalgesia treatment, subject received suggestions of increased pain unpleasantness, i.e., to make a visual imagine of an unpleasant situation in which her right median finger was hold in a vise that was enhancing the grip with the flowing of time of painful stimulation: *"*… *Although you will continue to experience normal sensation*, *your experience will seem more unpleasant*,. *as if your right middle finger is hold in a vise that is enhancing the grip*,. *more painful and uncomfortable*,. *now the vise is enhancing the grip at its maximum level*. *It is more disturbing than you might have expected*…*"*. For hypoalgesia treatment, the subject was suggested to produce an obstructive hallucination of stimulus perception on the right middle finger: *"… When you feel the unpleasant stimulus on your right middle finger*, *you may experience how much less intense the sensation is than you might have expected it to be*, *it can be as if your hand is covered by a plastic glove which makes the unpleasant stimulation more attenuated*,*…you can experience that all sensations in the finger will be attenuated…*. For control treatment, subject was suggested to feel feet, legs, arms, and hands deeply relaxed, the body more tired, sleepy, heavy, the breathing freely and deeply: *"… You are relaxed*. *relaxed*. *deeply relaxed*. *When you think to be relaxed*, *your muscles will relax*. *You are going to reach a state of deeper*, *whole relaxation… May be you are feeling that a pleasant warm tingle is spreading throughout your body and you are getting more and more tired and sleepy*. *sleepy… drowsy… and sleepy*. *You feel numb or heavy in your legs and feet*,*… in your hands*, *arms and shoulder*. *All your body is getting heavy*,. *heavy*, *and you are so heavy that you feel sinking into the chair… Your breathing is slow and regular*,. *slow and regular*. *You can feel your breath flowing slowly and deeply*,. *slowly and deeply"*. Hyperalgesia, hypoalgesia, and control tasks were performed with eyes-closed in both waking and hypnosis conditions. Following each suggestion, 120 pain stimuli were presented over a 132 sec period resulting in 120 trials of EEG data. After each recording session, subjective pain experiences were rated. At the end of pain treatments, the subject was waked up from hypnosis. Both waking and hypnosis conditions and the order of treatments within each condition were counterbalanced across subjects in order to avoid possible order effects or habituation. Between waking and hypnosis conditions, a resting period of 10 min was given. Each treatment condition lasted about 3 min (.8 min for suggestion instruction and 2.2 min of painful stimulation). At the end of each treatment, subjects were asked to rate both pain intensity and distress intensity, respectively as measures of the sensory-discriminative and affective-motivational components of pain, by using two separate 10 point numerical rating scales (NRS) [72]. The order of pain and distress intensity ratings was counterbalanced across subjects. The NRS-sensory scale displayed respectively on the left and right sides the descriptors‘0 = no pain sensation’ and ‘10 = the most intense pain sensation imaginable’. A similar rating scale was used for NRS-distress, the descriptors ranged from ‘0 = not at all distressful’ to ‘10 = the most distress imaginable’. The distinction between pain intensity (i.e., how intense was the sensation) and distress intensity (i.e., as annoying and unpleasant was the sensation), and between the two scales, was defined before the experiment started. To maintain the subjects’ attention and expectation high, the suggestion protocol relative to each treatment was repeated two times, i.e., after each minute of electric stimulation.

### EEG recording

EEG and electro-ocular (EOG) activities were acquired continuously and simultaneously with the performance measures by using a 40-channel NuAmps DC amplifier system (Neuroscan Inc.), set at a gain of 200, sampling rate of 512 Hz, and with signals band-limited to 100 Hz. Data were recorded and stored on a computer running Neuroscan Acquire 4.3 software. Electrode impedance was lower than 4 kΩ. The horizontal EOG was monitored via a pair of tin electrodes placed 1 cm lateral to the outer canthus of each eye and the vertical EOG was monitored via a separate bipolar montage placed above and below the center of the left eye. EEG data were recorded from 30 scalp sites (Fp1, Fp2, F7, F8, F3, F4, FT7, FT8, T3, T4, FC3, FC4, C3, C4, CP3, CP4, TP7, TP8, T5, T6, P3, P4, O1, O2, Fz, FCz, Cz, CPz, Pz, Oz) using a pure-tin electrode electrocap and referenced to digitally linked ears [(A1 + A2)/2] using Neuroscan Acquire setting. The ground electrode was located 10 mm anterior to Fz. The EEG was later processed by using Brain Vision Analyzer system (Brain Product). During a pre-processing stage, each signal was first applied a digital 50 Hz notch filter and then a bandpass filter (.5–70 Hz). The EEG was then reconstructed into discrete, single-trial epochs. For each stimulus, an EEG epoch length of 900-ms was used with a 150-ms pre-stimulus baseline and a 750-ms time window following the onset of the electric pulse. Epochs were rejected from averaging if amplitude exceeded ±75 μV, and eye blinks were corrected using Gratton’s et al.’s procedure [73]. Additional movement artifacts were removed manually. No participants had less than 100 accepted trials for averaging in any condition. All SERP data were baseline corrected. A SERP response was obtained for each experimental treatment in waking and hypnosis condition. This was done with the aim to elicit a more stimulus oriented SERP response i.e., the N140 wave [24a, 24b], and a more specific cognitive P200 wave (a component of the P300 family waves) reflecting pain-related cognitive-attention processing [74–76].

In line with previous reports [48–49], two reliable SERP components were observed. The N140 wave (peak latency: M = 140.5, SD = 6.2 ms) was fronto-centrally distributed, and quantified as the minimum peak amplitude detected within a 130–160 ms time window. The P200 wave (peak latency: M = 22.4, SD = 3.8 ms) was fronto-central to centro-parietal distributed peaking at about 220 ms. This was labeled as P200 and scored as the most positive peak in the waveform, observed within a 200–250 ms time window after stimulus onset.

### Statistical analyses

A simple ANOVA with hypnotizability, serving as between group variables, was used to assess individual differences on subjective threshold measures.

To test the impact of hypnotizability on responses to hypoalgesia and hyperalgesia suggestions during waking and hypnosis condition, pain and distress rating measures were submitted to analysis of variance (ANOVA; SAS-9.2, glm procedure) with Hypnotizability (HHs vs LHs) as between subjects factor and Condition (waking, hypnosis) and Suggestion (control, hypoalgesia, hyperalgesia) as within subjects factor.

For each N140 and P200 peak amplitude measures, a repeated measures ANOVAs was carried out according to the following design: 2 Condition (Waking, Hypnosis) x 3 Treatment (Control, Hypoalgesia, Hyperlagesia) x 3 Head Level (Left, Mid, Right) x 5 Location (Frontal, Fronto-central, Central, Centro-parietal, Parietal). Contrasts F values were also obtained when necessary. To prevent the risk of falsely significant results, as may happen using repeated measures ANOVAs if the sphericity assumption has been violated [77], the Huynh-Feldt epsilon correction of significance levels was applied when necessary. Post hoc comparisons were carried out by using a t-test procedure with Bonferroni correction of α =. 05 [78].

### LORETA method for cortical sources analysis of the N140 and P200 waves

SERP responses were exported for further analysis using sLORETA software provided by the KEY Institute for Brain-Mind Research (University Hospital of Psychiatry, Zurich, Switzerland; http://www.uzh.ch/keyinst/NewLORETA/LORETA01.htm). This was done since sLORETA software has been found useful for the analysis of different time segments of ERPs [17]. sLORETA enables the spatial identification and analysis of brain cortical activity via conventional EEG recordings [59, 79–81]. sLORETA performs source localization in 6239 cortical gray matter voxels sized 5 mm^3^ rather than 7 mm^3^ offered by the previous LORETA version, and localization inference is based on standardized values of the current density estimates [82]. The solution space of sLORETA is restricted to cortical and some hippocampal and amygdala gray matter defined via a reference brain from the Brain Imaging Center at the Montreal Neurological Institute (MNI) [83,84]. The sLORETA implementation incorporates a 3-shell spherical head model registered to a recognized anatomical brain atlas [85]. Individual 3-D electrodes are positioned by the Talairach coordinate system according to the spatial association between anatomical brain landmarks and scalp positions [86].

For source reconstruction, the subtractions of SERP traces between painful stimuli of hyperalgesia, hypoalgesia, and control treatments, as well as between high hypnotizable and low hypnotizable subjects, were assessed using sLORETA within time intervals of 130–160 ms, 200–250 ms respectively for the N140, and P200 waves. sLORETA enables the computation of statistical maps from EEG data that indicate the locations of the underlying source processes with low error [87]. Statistical significance was assessed using a non-parametric randomization test defined by 5000 randomizations [87]. No transformations or normalizations were used for calculated source data before statistical analyses. sLORETA maps were statistically analyzed using the paired Student *t* test for treatments’ comparisons and independent groups *t* test (t-threshold set to p <. 05). At the cluster level, all voxels showing a statistically significant difference were detected. For descriptive issues, the Talairach coordinates (TCs) of local maxima for the statistical comparison have been reported in the text. It is important to note that this localization is not a complete listing of all significantly different cortical areas, but a listing of the local maxima of these differences. Electrophysiological data analyses have been banked in our lab archive and are available upon request.

### Regions of interest (ROIs) to electric stimulation using sLORETA and correlational analyses

Based on estimated electric current density (μA/mm^2^), differences in activity between each treatment of noxious stimulation and a resting-baseline stimulation-free, in waking and hypnosis conditions, were statistically assessed using nonparametric permutation test with 5000 randomizations accounting for multiple voxel-by-voxel comparisons implemented in sLORETA software. Maps were statistically analyzed using the sLORETA paired Student *t* test (threshold p<.05). All voxels showing a statistically significant amplitude increase in response to noxious stimuli, compared with resting baseline, were detected. Clusters of significant activation were defined as ROIs and included the middle frontal gyrus (BA6), postcentral gyrus (BA3), and anterior cingulate (BA24) within a time window of 190–270 ms. For further activation magnitude analysis, each ROI was characterized by a single-voxel spatial local maximum. To accept a spatial local maximum, the thresholds of the *t* values in the particular voxel had to be greater compared with all surrounding voxels. All voxel locations are given according to the Talairach coordinate. For each treatment and condition, current density waveforms of each ROI were obtained at 512 Hz in a time window of 450 ms from stimulus onset, yielding high-resolution temporal curves. For further activation magnitude analysis, each ROI was characterized by a single-voxel local spatial maximum. Subsequently, areas under the curves (AUCs) in a neighborhood of the maximum were calculated using a 25 ms time window and then correlated with subjective pain and distress ratings obtained during each treatment in both waking and hypnosis conditions. Current density scores of ROIs showing significant Bonferroni's corrected correlations with subjective numerical pain and distress scores were used in multiple regression analyses as predictors of pain/distress scores. These analyses were mainly performed to highlight brain responses coding for pain/distress and also served to evaluate if the brain pattern of pain/distress coding may change depending on the experimental treatment and condition. We first entered as predictors in the regression model (Step-1) the BAs density scores that were significantly correlated with pain (or distress) scores (Table 2). Subsequently, hypnotizability was entered in the model (Step-2) to test the effect of this variable in the prediction.

To examine, in a more direct way, how pain modulation relates to brain activity, we performed separate multivariate regression analyses for each treatment and condition including difference scores of current density as predictors and pain rating difference scores as a dependent variable. Difference scores were calculated by subtracting scores obtained for hypoalgesia from those for control treatment (Control vs Hypoalgesia), scores obtained for control from those for hyperalgesia (Hyperlgesia vs Control) and, to optimize leverage in the analysis (i.e., the largest differences in pain), scores obtained for hypoalgesia were subtracted from those obtained for hyperalgesia treatment (Hyperalgesia vs Hypoalgesia). In addition, hypnotizability was entered in the model as a potential mediator of the relation between pain modulation and brain activity.

## Results

### Pain and distress ratings

Since recent findings indicate that highly hypnotizable individuals are distributed across two classes of response patterns, one suggesting an inward attention subtype and the other a dissociative subtype [88], there is reason to assume that there could be at least two different HH groups which may differ in attentional control. In order to treat HH and LHs as homogeneous normally distributed groups, we applied Shapiro-Wilk test on pain and distress ratings responses in both waking and hypnosis conditions. This test did not disclose violations of the assumptions of homogeneity of variance, separately within each HH and LH group, for both pain and distress scores in both waking and hypnosis conditions, the rejection of the null hypothesis of normality was far from the significance level (.893 < W <. 981, p values ranged from. 06 to. 970).

**Fig 1:**
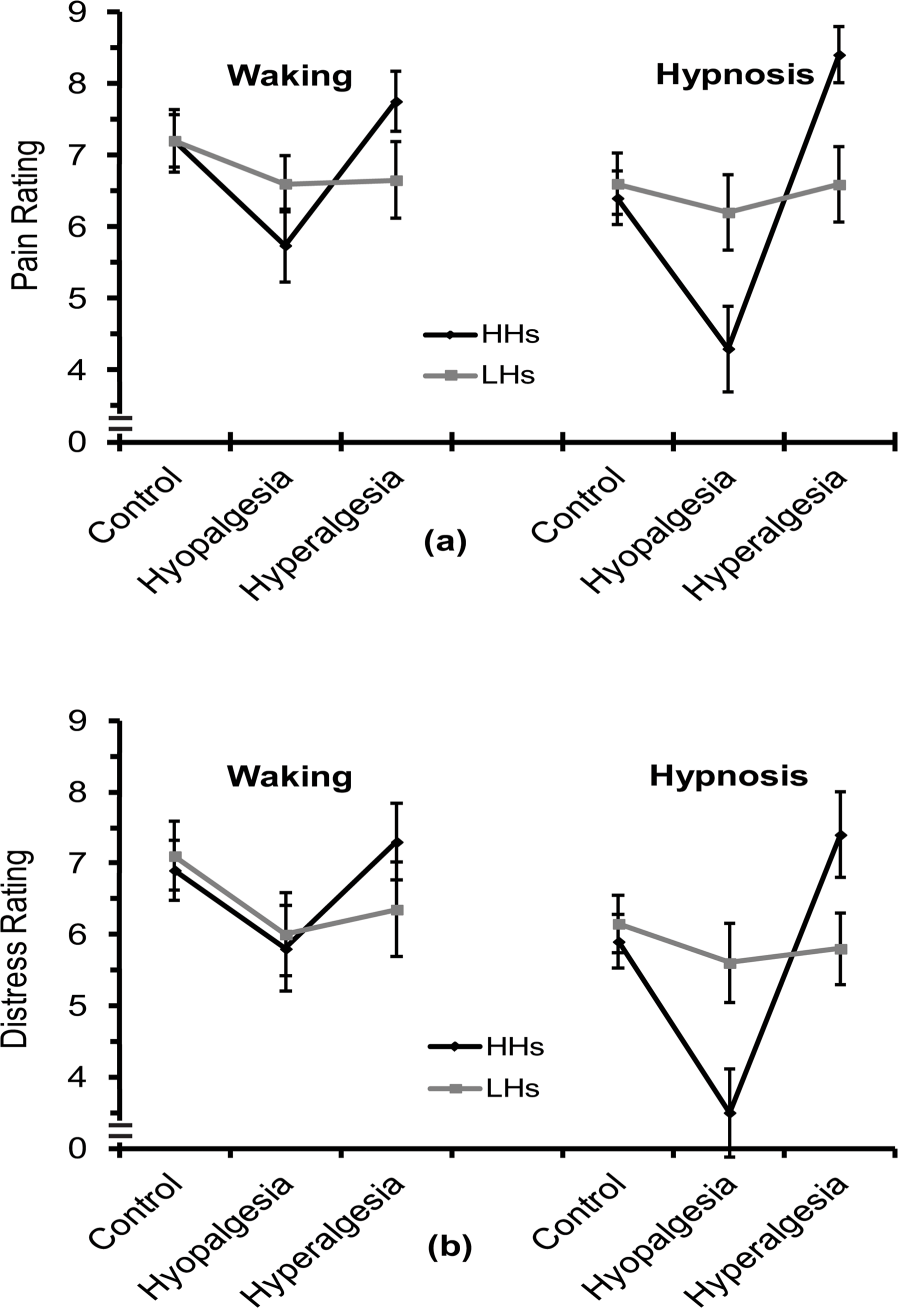
Mean and standard errors of pain, panel (a) and distress ratings, panel (b), respectively as measures of sensory-discriminative and affective-motivational components of pain, in high and low hypnotizable subjects (HHs and LHs). Measures were obtained during three treatments (Control, Hypoalgesia, and Hyperalgesia) in waking and hypnosis conditions.

As indicated by the statistics in Table 1, the ANOVA on pain rating scores failed to show significant differences between HH and LH groups and between waking and hypnosis conditions. However, the main effect for Treatment and the interaction of Hypnotizability with Treatment were significant (see Table 1). The first effect indicated that, in comparison with relaxation-control, pain sensation was reduced for analgesia treatment (post-hoc t-test, control vs hypoalgesia: t = 5.5, p <. 0001) and enhanced for hyperalgesia treatment (control vs hyperalgesia: t = -2.4, p <. 05; see Fig 1A). The interaction effect indicated that in HHs, hyperalgesia and hypoalgesia treatments had significant pain reduction and pain enhancement (control vs hypoalgesia: t = 7.6, control vs hyperalgesia: t = -11.1), while for LHs, there were no significant differences between treatments (all ts < 1; Fig 1A). Moreover, the interaction of Treatment with Condition, and the triple interaction between these two factors with Hypnotizability were both significant (Table 1). These significant effects indicated that in the HH group, either for the waking and hypnosis, both treatments of hypoalgesia and hyperalgesia were effective in the modulation of pain, although in the condition of hypnosis both treatments produced the highest changes in pain sensation (HHs—waking: control vs hypoalgesia, t = 7.6, p <. 0001 control vs hyperalgesia: t = -3.1, p <. 05; HHs—hypnosis: control vs hypoalgesia, t = 5.9, p <. 001, control vs hyperalgesia, t = -1.8, p <. 0001). These effects were not observed in LHs during hypnosis (LHs: all t-tests were not significant, p >. 05; see Fig 1A). However, in LHs during waking condition there were significant pain reductions for both hypoalgesia and hyperalgesia compared to control treatment (LHs—waking: control vs hypoalgesia: t = 3.1, p <. 05, control vs hyperalgesia: t = 3.9, p <. 01; Fig 1A).

**Table 1:** Statistics for Pain Rating, Distress Rating, N140 and P220 peak amplitudes.

		Pain Rating		Distress Rating		N140 Amplitude		P220 Amplitude	
Effect	df	F	p	F	p	F	p	F	p
**Hy: Hypnotizability**	1, 18								
**C: Condition**	1, 18			25.66	<.0001	4.78	<.05		
**T: Treatment**	2, 36	72.18	<.0001	73.83	<.0001				
**T x Hy**	2, 36	51.91	<.0001	41.99	<.0001	6.39	<.05		
**C x T**	2, 36	11.6	<.0001	11.6	<.001			25.65	<.0001
**C x T x Hy**	2, 36	5.26	<.01	15.03	<.0001	6.31	<.01	5.4	<.01
**HL: Head Level**	2, 36					4.66	<.05	26.44	0.0001
**Lo: Location**	4, 72					3.1	<.05	13.68	<.0001
**C x T x HL**	4, 72							3.89	<.01
**C x T x HL x Hy**	4, 72							2.34	<.05
**C x T x Hy x Lo**	8, 144					3.02	<.01		

The ANOVA on distress rating scores showed a main effect for Condition and for Treatment (Table 1). The first effect indicated that distress was significantly reduced during hypnosis as compared to waking condition (Fig 1B). The second effect disclosed that, in comparison with relaxation-control treatment, there was a reduced distress score for hypoalgesia (control vs hypoalgesia: t = 7.9, p <. 0001, Fig 1B), while for hyperalgesia there was no significant difference in distress compared to control (t<1, p >. 05). Moreover, the significant interaction of Treatment with Condition (Table 1) indicated that, during hypnosis, hypoalgesia treatment produced a significantly distress reduction, and hyperalgesia a significant distress increase, while, during waking, only a significant distress reduction was obtained for hypoalgesia (hypnosis: control vs hypoalgesia t = 5.1, p <. 0001, control vs hyperalgesia, t = -2.5, p <. 05; waking: control vs hypoalgesia t = 8.3, p <. 0001, control vs hyperalgesia, t = -.9, p>.05; Fig 1B). Finally, we found a significant second order interaction of Treatment with Hypnotizability, and a significant third order interaction of these two factors with Condition (Table 1). These effects indicated that, in comparison with control treatment, HHs, for both wake and hypnosis conditions, were able to reduce and enhance distress sensation during hypoalgesia and hyperalgesia, although during hypnosis both treatments produced a more pronounced modulation of distress sensation in the expected direction (HHs—waking: control vs hypoalgesia: t = 7.6, p <. 0001, control vs hyperalgesia: t = -3.1, p <. 05; HHs—hypnosis: control vs hypoalgesia: t = 5.9, p <. 001, control vs hyperalgesia: t = -1.8, p <. 0001 and hypoalgesia vs hyperalgesia t = -9.1, p <. 0001; Fig 1B). These effects were not observed for LHs during hypnosis (all t-tests were under the level of significance; Fig 1B), but these subjects, during waking, disclosed a significant reduction of distress for hypoalgesia and hyperalgesia treatments (LHs—waking: control vs hypoalgesia: t = 4.7, p <. 01, control vs hyperalgesia: t = 3.0, p <. 05; Fig 1B).

### Effects of experimental factors on N140 and P200 peak amplitudes

**Fig 2:**
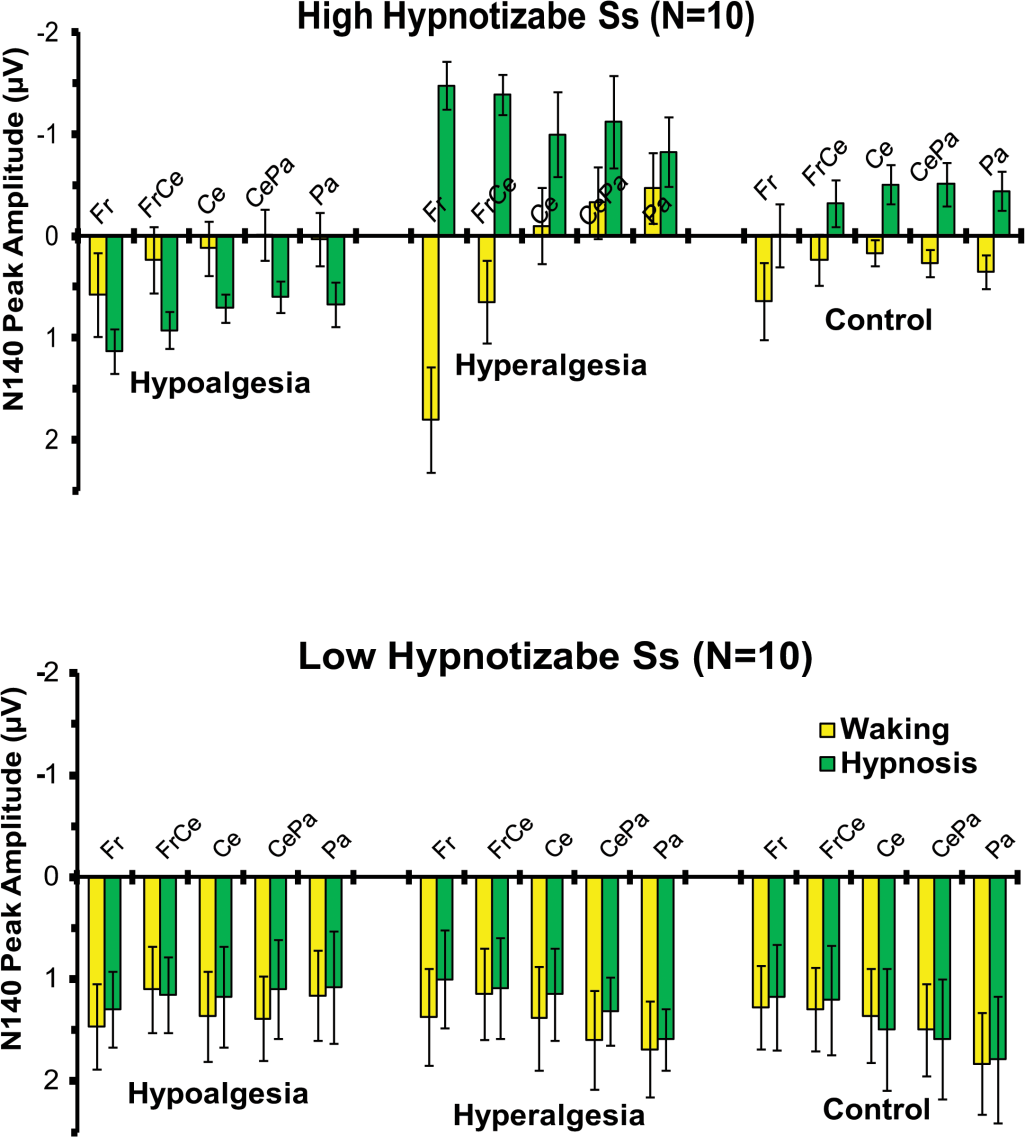
Mean amplitudes and standard errors of N140 peak amplitude (negative values) averaged across frontal (Fr), frontocentral (FrCe), central (Ce), centroparietal (CePa) and parietal (Pa) locations for the Hypoalgesia, Hyperalgesia, and Control treatments during waking and hypnosis conditions in high (top panel) and low (bottom panel) hypnotizable subjects (HHs and LHs). The histogram in the top clearly shows that in HHs, during hypnosis, hyperalgesia, compared to the control treatment, induced an enhanced N140 negative peak at frontal, frontocentral, central, centroparietal, and parietal sites, while hypoalgesia had reduced N140 wave (positive values). The bottom panel shows that LHs did not exhibit significant N140 amplitude differences between treatments in both waking and hypnosis conditions

**Fig 3:**
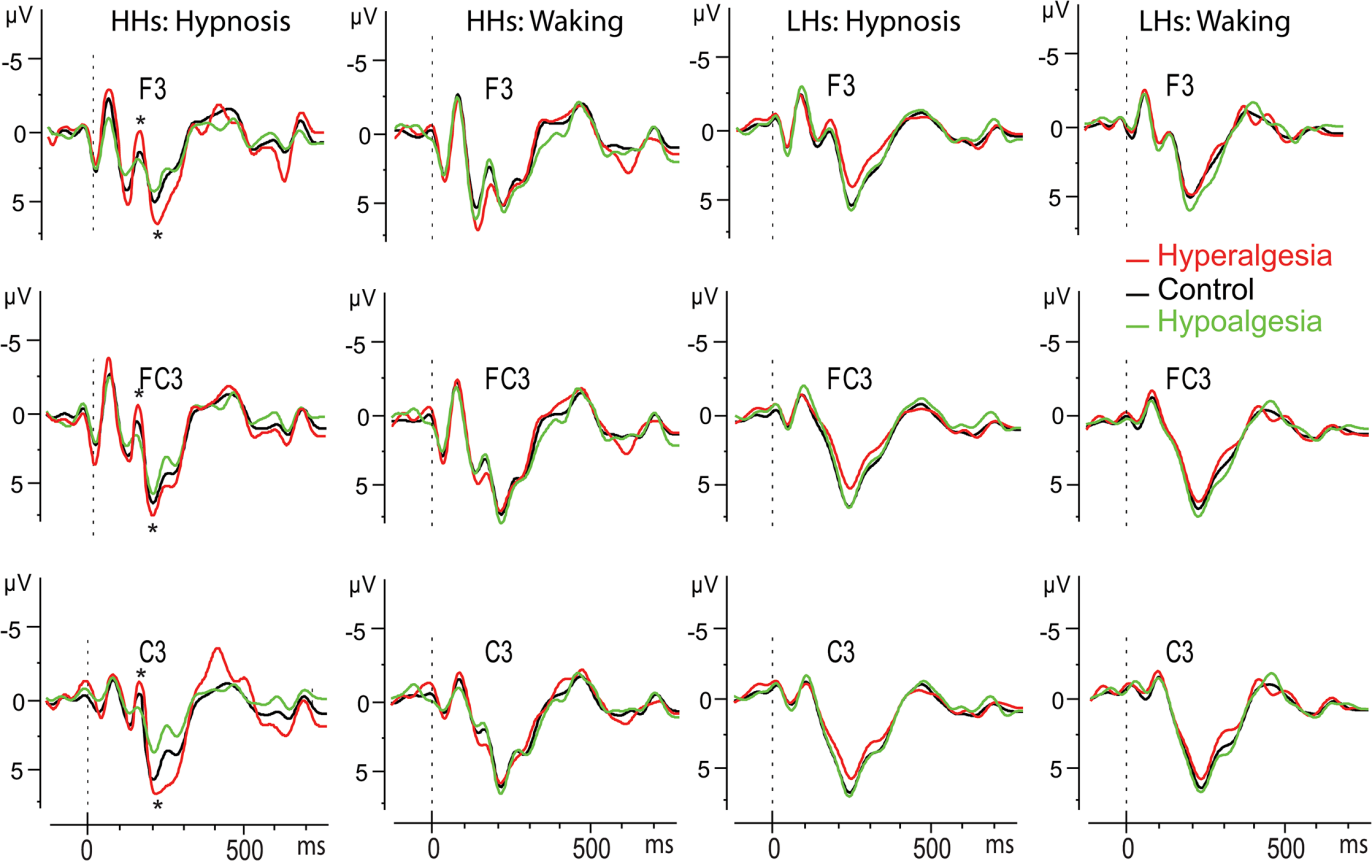
SERPs of the most sensitive scalp sites (F3, FC3, and C3). Significant peak differences between treatments for the N140 and P200 waves are displayed in the left side (*, p<.05).

**Fig 4:**
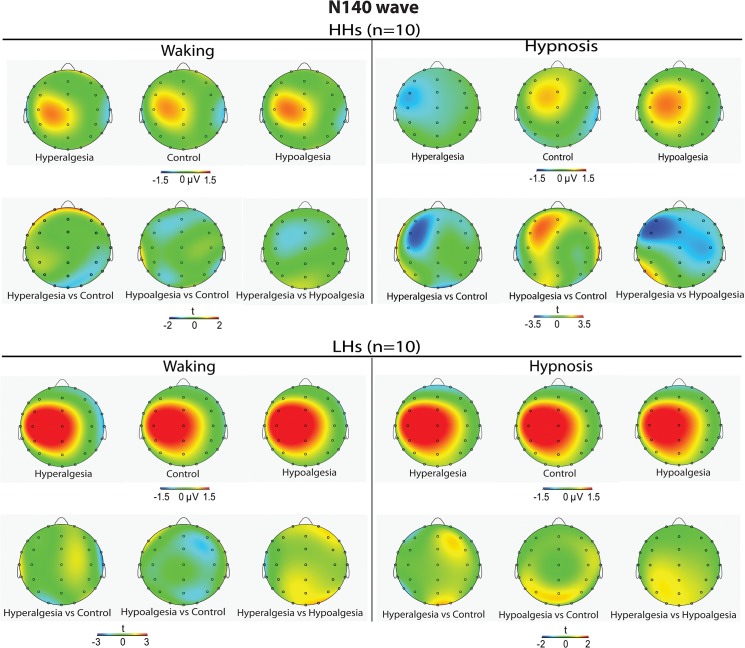
Averaged scalp topography of N140 wave (peaking at 140.5±6.2 ms within a time window of 130–160 ms) for Hyperalgesia, Control, and Hypoalgesia treatments in high (top-panel, first row) and low (bottom-panel, first row) hypnotizable participants (HHs and LHs) during waking (left panel) and hypnosis (right panel) conditions. t-Test maps comparing the three treatments are shown in the second row of each top and bottom panel.

As shown by the statistics in Table 1, the ANOVA performed across N140 amplitudes (negative values) yielded significant main effects for Condition and Head Level, indicating greater negativity during hypnosis than waking condition (-.25 vs. 43 μV, respectively), and greater negativity in the left head sides with respect to the center and right ones (-.16 vs. 34 and. 12 μV). The Treatment by Hypnotizability and Treatment by Hypnotizability by Condition, and Condition by Treatment by Hypnotizability by Location interactions were also all significant (Table 1). The last effect is displayed in Fig 2, wherein is clearly depicted that in HHs, during hypnosis, hyperalgesia, compared to the control treatment, produced an enhanced N140 negative peak at frontal, frontocentral, central, centroparietal, and parietal sites, while hypoalgesia had reduced N140 wave. In contrast, LHs did not exhibit significant N140 amplitude differences between treatments in both waking and hypnosis conditions (see bottom panel of Fig 2). SERPs of the most sensitive scalp sites (F3, FC3 and C3) are reported in Fig 3. Topographic head-maps of the N140 wave (130–160 ms) and t-test statistic maps for comparisons across treatments are depicted in Fig 4. Here is clearly shown that the above mentioned effects were mainly involving the left hemisphere.

**Fig 5:**
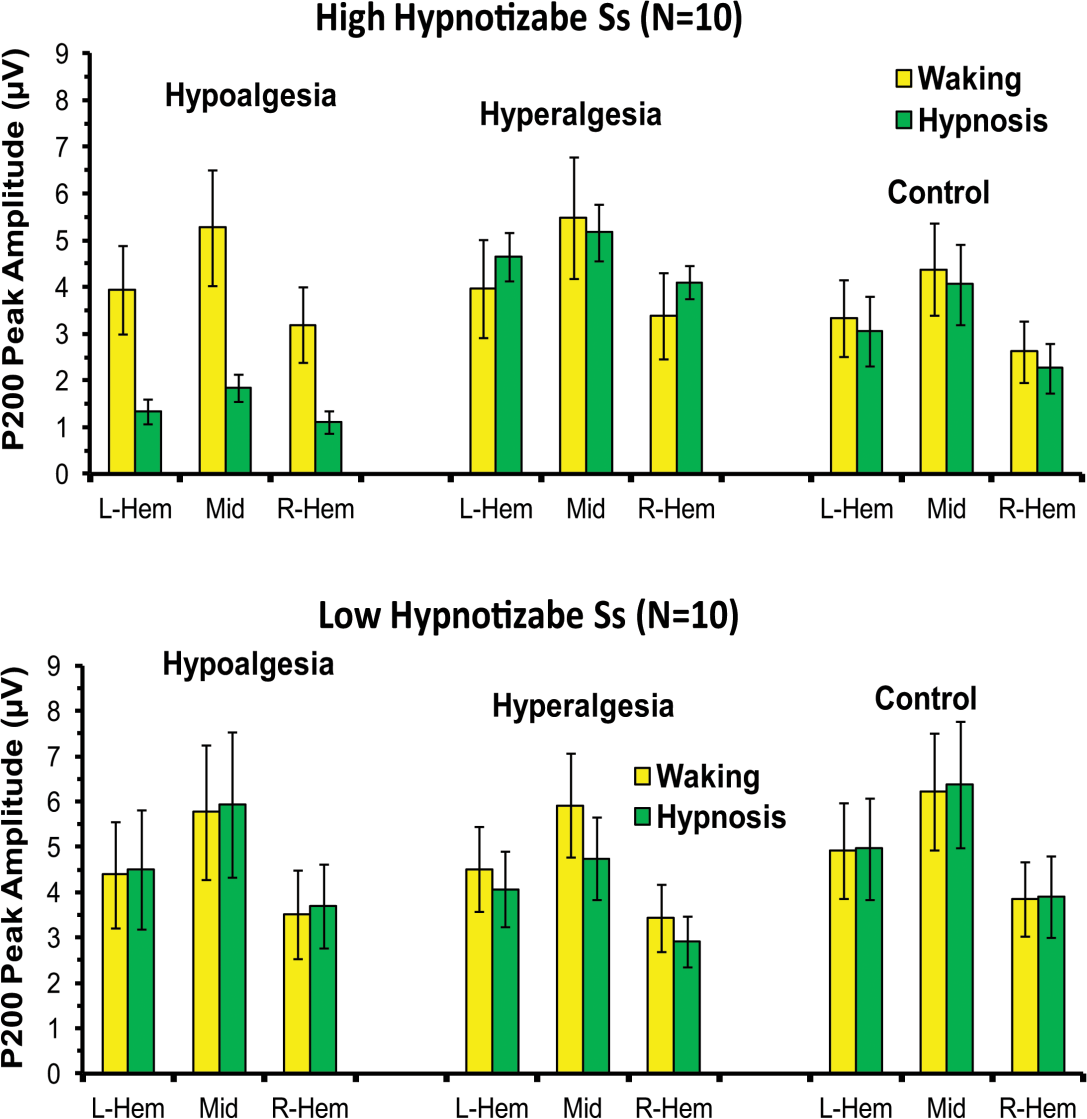
Mean amplitudes and standard errors of P200 peak amplitude averaged across left and right-hemisphere and medium head side (L-Hem, R-Hem, and Mid) for the Hypoalgesia, Hyperalgesia, and Control treatments during waking and hypnosis conditions in high (top panel) and low (bottom panel) hypnotizable subjects (HHs and LHs). Both top and bottom histograms clearly shows larger positive peaks in the midline compared to the left and right sides as well as in the midline compared to right side. The top panel shows that in HHs, the hyperalgesia treatment during hypnosis had higher P200 amplitudes than control treatment, while hypnotic hypoalgesia had smaller peaks. These differences disappeared in waking condition. The bottom panel shows that LHs did not exhibit significant P200 amplitude differences between treatments in both waking and hypnosis conditions

**Fig 6:**
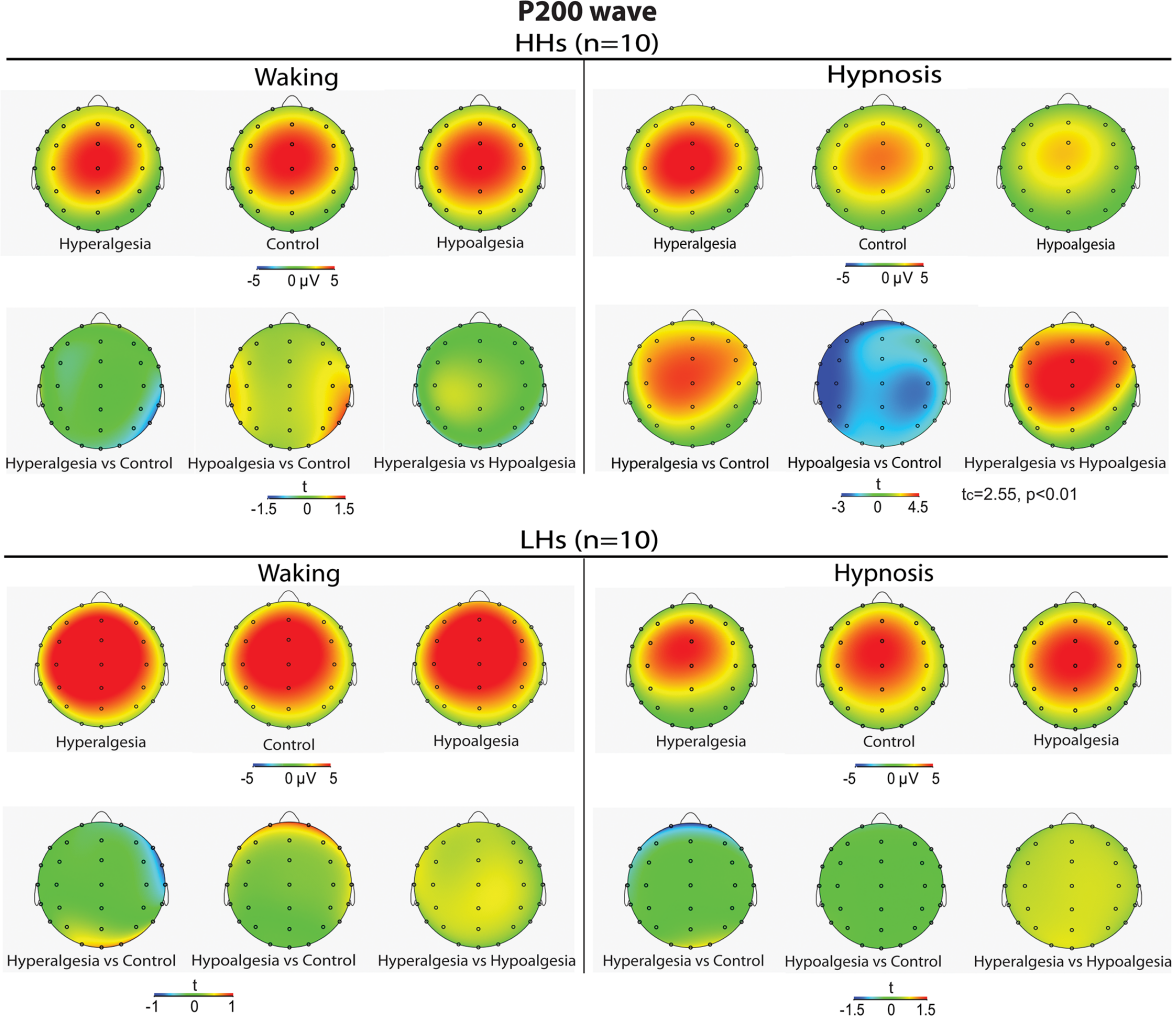
Averaged scalp topography of P200 wave (peaking at 220.4±3.8 ms within a time window of 200–250 ms) to Control, Hyperalgesia, and analgesia treatments in high (top-panel, first row) and low (bottom-panel, first row) hypnotizable participants (HHs and LHs) during waking (left panel) and hypnosis (right panel) conditions. t-Test maps comparing the three treatments are shown in the second row of each top and bottom panel.

The ANOVA on P200 amplitudes disclosed two significant main effects, one for Head Level and the other for Location (Table 1). The first effect showed larger positive peaks on the midline and left head sides compared to right sides (see Fig 5). The second effect disclosed greater positivity (p <.001) at frontocentral and central sites compared to the other scalp sites (3.1, 4.2, 5.0, 5.0, 4.2, 3.3 μV, for prefrontal, frontal, frontocentral, central, centroparietal, and parietal regions). In addition, the following interactions were also significant: (1) Condition by Treatment, (2) Condition by Treatment by Hypnotizability, (3) Condition by Treatment by Head Level, and (4) Condition by Treatment by Hypnotizability by Head Level (Table 1 and Fig 5). These effects, in the whole, showed that in hypnosis condition the HHs had, in comparison with control treatment, enhanced P200 amplitudes (mainly across midline and left head sites) during hyperalgesia and reduced peaks during hypoalgesia, while these differences disappeared in waking condition. In contrast, LHs did not exhibit significant P200 amplitude differences between treatments in both waking and hypnosis conditions (Figs 3, 5 and 6). Differences for P200 SERP of the most sensitive left scalp sites are shown in Fig 3. Topographic head-maps of the P200 wave (200–250 ms) and t-test statistic maps for comparisons across treatments are depicted in Fig 6. Here is clearly depicted that this SERP wave is frontocentral distributed.

### Correlation analyses of pain and distress coding across treatments using sLORETA localization

**Fig 7:**
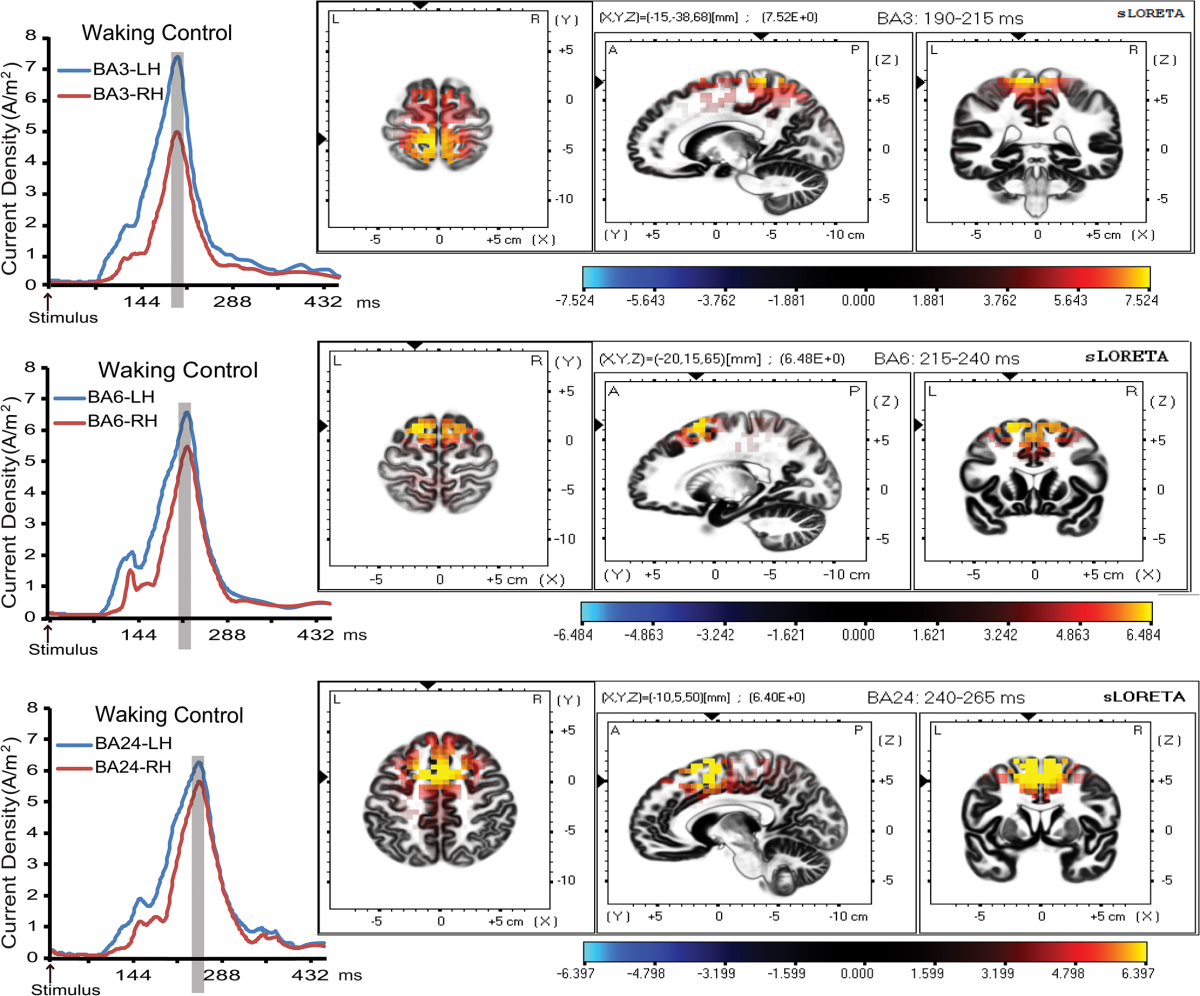
Significant activation waveforms of LORETA current source density (left) and anatomical maps (right) of the spatial local maximum of BA3, BA6, and BA24 pain-related cortical regions in both left and right hemisphere (LH and RH) after application of noxious electric pulses to the right middle finger, during waking-relaxation control treatment (coordinates are shown in Table 2).

Source estimation analysis revealed significant activation of BA3, BA6, and BA24 (coordinates are shown in Table 2) in response to noxious electric stimulus. Since there were no waveform differences between temporal activation profiles and anatomical sLORETA modeling of each pain related regions of interest (ROI) across treatments in waking and hypnosis conditions, only waveforms and sLORETA anatomical maps for the waking-control treatment are depicted in Fig 7. In waking condition, the correlations between pain ratings and current density at BA3, BA6 and BA24 contralateral to the stimulation side, were the only ones that remained significant after Bonferroni correction for each treatment (see Table 2). During hypnosis condition, higher activations in the right and left BA3 region were respectively associated with increased pain ratings in the control and hyperalgesia treatments, while for hyperalgesia an enhanced activation in both hemispheres at BA3 was associated with increased pain scores (Table 2).

**Table 2:** Correlations of pain and distress ratings with the area under the curve of cortical Pain-ROIs of current densities to noxious electric stimuli applied to the middle finger of the right hand across all subjects (N = 20).

				Waking Control	Waking Hypo-algesia	Waking Hyper-algesia	Hypnosis Control	Hypnosis Hypo-algesia	Hypnosis Hyper-algesia
			
			
BA Region	X	Y	Z						
Pain Rating				r	r	r	r	r	r
**BA3-LH**	-20	18	59	0.75**	0.75**	0.79**	0.52	0.74**	0.59*
**BA3-RH**	15	-34	64	0.45	0.49	0.4	0.58*	0.70**	0.55
**BA6-LH**	-15	-34	64	0.65*	0.47	0.59*	0.47	0.46	-0.16
**BA6-RH**	20	18	59	0.36	0.17	0.53	0.46	0.44	-0.21
**BA24-LH**	-10	7	46	0.69**	0.41	0.43	0.46	0.44	-0.19
**BA24-RH**	10	7	46	0.60*	0.27	0.56	0.46	0.44	-0.2
**Distress Rating**									
**BA3-LH**	-20	18	59	0.58*	0.47	0.69**	0.46	0.68**	0.48
**BA3-RH**	15	-34	64	0.46	0.18	0.37	0.53	0.75**	0.45
**BA6-LH**	-15	-34	64	0.41	0.34	0.53	0.49	0.55	0.11
**BA6-RH**	20	18	59	0.31	0	0.51	0.48	0.54	0.08
**BA24-LH**	-10	7	46	0.64*	0.4	0.41	0.48	0.5	0.09
**BA24-RH**	10	7	46	0.56	0.25	0.56	0.48	0.54	0.09

Abbreviations: BA Brodmann's area; BA6, middle frontal gyrus (frontal lobe); BA3, postcentral gyrus (parietal lobe, primary somatosensory cortex); BA24, anterior cingulate (limbic lobe); r, correlation coefficient (* p<.05, ** p<.01, after Bonferroni's correction). Locations are according to the Talairach coordinate system (x, mediolateral; y, rostrocaudal; z, dorsal-ventral).

* p<.05

** p<.01

The results of the regression analyses for waking and hypnosis condition are presented in Tables 3 and 4. Findings for pain and distress ratings are displayed, respectively, in the left and right half of each of these tables. In waking condition, regression analyses for pain rating revealed a consistent effect across treatments showing that current density at BA3 contralateral to the stimulated hand is a stable predictor of pain felt. Moreover regressions in Step-2 indicated that adding hypnotizability in the model did not account for significant changes in the observed significant relationship, with the exception of hyperalgesia treatment wherein higher hypnotizability was significantly (p = 0.021) associated with increased pain perception (see bottom of Table 3). Regression analyses using distress rating as a dependent variable showed that during the control treatment the best predictor was the activation level of BA24 in the left hemisphere. Since we failed to find significant correlations for hypoalgesia (see Table 2) no regressions were performed for this treatment. During hyperalgesia treatment the current density at BA3 contralateral to the stimulated hand was found to be a good predictor of distress felt, and hypnotizability level was a significant mediator of this relationship.

**Table 3:** Multivariate regression analysis using area under the curve of pain-related BA3, BA6 and BA24 activation in the left hemisphere as predictors of pain and distress ratings in Waking condition (step-1) and after entering SHCS scores as a covariate (step-2).

Pain Rating					Distress Rating			
BA Region	Estimate	SE	t	P	Estimate	SE	t	P
**Control** ^1^					**Control** ^7^			
**Intercept**	4.971	0.509	9.76	<.0001	5.371	0.466	11.53	<.0001
**BA3-LH**	0.207	0.068	3.04	0.008	0.106	0.071	1.49	0.1542
**BA24-LH**	0.052	0.35	0.15	0.884	0.459	0.199	2.3	0.034
**BA24-RH**	0.52	0.5	1.04	0.314	-	-	-	-
**BA6-LH**	0.008	0.108	0.07	0.943	-	-	-	-
**Control** ^2^					**Control** ^8^			
**Intercept**	4.53	0.638	7.11	<.0001	5.079	0.725	7	<.0001
**SHCS**	0.19	0.169	1.12	0.281	0.095	0.177	0.54	0.599
**BA3-LH**	0.237	0.072	3.26	0.006	0.121	0.078	1.55	0.14
**BA24-LH**	0.251	0.39	0.64	0.531	0.447	0.205	2.18	0.044
**BA24-RH**	0.24	0.555	0.43	0.673	-	-	-	-
BA6-LH	-0.034	0.113	-0.3	0.767	-	-	-	-
**Hypoalgesia** ^3^					**Hypoalgesia** ^9^			
**Intercept**	5.26	0.289	18.21	<.0001	-	-	-	-
**BA3-LH**	0.196	0.041	4.77	0.0002	-	-	-	-
**Hypoalgesia** ^4^					**Hypoalgesia** ^10^			
**Intercept**	5.67	0.548	10.34	<.0001	-	-	-	-
**SHCS**	-0.15	0.171	-0.88	0.391	-	-	-	-
**BA3-LH**	0.19	0.042	4.47	0.0003	-	-	-	-
**Hyperalgesia** ^5^					**Hyperalgesia** ^11^			
**Intercept**	5.055	0.454	11.13	<.0001	5.57	0.364	15.29	<.0001
**BA3-LH**	0.26	0.065	3.98	0.001	0.213	0.052	4.1	0.0007
**BA6-LH**	0.158	0.116	1.36	0.191	-	-	-	-
**Hyperalgesia** ^6^					**Hyperalgesia** ^12^			
**Intercept**	4.213	0.514	8.19	<.0001	4.683	0.463	10.11	<.0001
**SHCS**	0.352	0.138	2.55	0.021	0.34	0.13	2.62	0.0179
**BA3-LH**	0.277	0.057	4.85	0.0002	0.219	0.045	4.85	0.0001
**BA6-LH**	0.121	0.102	1.19	0.251	-	-	-	-

^1^ Step-1: F(4,19) = 8.79, p = 0.0007; R-Square = 0.70, Adj. R-Square = 0.62

^2^ Step-2:Entering SHCS: F(5,19) = 7.41, p = 0.0014; R-Square = 0.72, Adj. R-Square = 0.63

^3^ Step-1:F(1,19) = 22.71, p = 0.0002; R-Square = 0.56, Adj. R-Square = 0.53

^4^ Step-2: Entering SHCS: F(2,19) = 11.60, p = 0.0007; R-Square = 0.58, Adj. R-Square = 0.53

^5^ Step-1: F(2,19) = 16.75, p<.0001; R-Square = 0.66, Adj. R-Square = 0.62

^6^ Step-2: Entering SHCS: F(3,19) = 16.94, p<.0001; R-Square = 0.76, Adj. R-Square = 0.71

^7^ Step-1: F(4,19) = 7.95, p = 0.0036; R-Square = 0.48, Adj. R-Square = 0.42

^8^ Step-2: Entering SHCS: F(3,19) = 5.18, p = 0.011; R-Square = 0.49, Adj. R-Square = 0.39

^9^ Non Significant

^10^ Non Significant

^11^ Step-1: F(1,19) = 16.81, p =. 0007; R-Square = 0.48, Adj. R-Square = 0.45

^12^ Step-2: Entering SHCS: F(2,19) = 14.58, p =. 0002; R-Square = 0.63 Adj. R-Square = 0.59

**Table 4:** Multivariate regression analysis using area under the curve of pain-related BA3, BA6 and BA24 activation in the left hemisphere as predictors of pain and distress ratings in Hypnosis condition (step-1) and after entering SHCS scores as a covariate (step-2).

Pain Rating					Distress Rating			
BA Region	Estimate	SE	t	P	Estimate	SE	t	P
**Control** ^1^					**Control** ^7^			
**Intercept**	5.301	0.44	12.05	<.0001	-	-	-	-
**BA3-RH**	0.215	0.071	3.04	0.007	-	-	-	-
**Control** ^2^					**Control** ^8^			
**Intercept**	5.154	0.606	8.51	<.0001	-	-	-	-
**SHCS**	0.055	0.152	0.36	0.721	-	-	-	-
**BA3-RH**	0.217	0.072	2.99	0.008	-	-	-	-
**Hypoalgesia** ^3^					**Hypoalgesia** ^9^			
**Intercept**	3.576	0.422	8.47	<.0001	3.033	0.401	7.56	<.0001
**BA3-LH**	0.408	0.19	2.15	0.046	0.193	0.181	1.07	0.3
**BA3-RH**	0.279	0.208	1.34	0.198	0.466	0.198	2.35	0.031
**Hypoalgesia** ^4^					**Hypoalgesia** ^10^			
**Intercept**	3.262	0.869	3.76	0.0017	3.572	0.815	4.38	0.0005
**SHCS**	0.097	0.231	0.42	0.682	-0.166	0.217	-0.76	0.457
**BA3-LH**	0.445	0.214	2.08	0.054	0.13	0.201	0.65	0.528
**BA3-RH**	0.268	0.215	1.24	0.231	0.485	0.202	2.4	0.029
**Hyperalgesia** ^5^					**Hyperalgesia** ^11^			
**Intercept**	5.447	0.718	7.58	<.0001	-	-	-	-
**BA3-LH**	0.272	0.087	3.12	0.006	-	-	-	-
**Hyperalgesia** ^6^					**Hyperalgesia** ^12^			
**Intercept**	4.22	0.604	6.99	<.0001	-	-	-	-
**SHCS**	0.621	0.151	4.1	0.0007	-	-	-	-
**BA3-LH**	0.229	0.065	3.55	0.002	-	-	-	-

^1^ Step-1: F(1,19) = 9.27, p = 0.007; R-Square = 0.34, Adj. R-Square = 0.30

^2^ Step-2 entering SHCS: F(2,19) = 4.48, p = 0.0274; R-Square = 0.34, Adj. R-Square = 0.27

^3^ Step-1: F(2,19) = 12.67, p = 0.0004; R-Square = 0.590, Adj. R-Square = 0.55

^4^ Step-2 entering SHCS: F(3,19) = 8.10, p = 0.0017; R-Square = 0.60, Adj. R-Square = 0.53

^5^ Step-1: F(1,19) = 9.70, p = 0.006; R-Square = 0.35, Adj. R-Square = 0.31

^6^ Step-2 entering SHCS: F(2,19) = 17.52, p<.0001; R-Square = 0.67, Adj. R-Square = 0.63

^7^ Non Significant

^8^ Non Significant

^9^ Step-1: F(2,19) = 12.36, p = 0.0005; R-Square = 0.59, Adj. R-Square = 0.54

^10^ Step-2: F(3,19) = 8.23, p = 0.0015; R-Square = 0.60, Adj. R-Square = 0.53

^11^ Non Significant

^12^ Non Significant;

Regression analyses during hypnosis condition yielded some changes in the brain pattern of pain or distress coding. During control treatment in hypnosis we disclosed that the activation level at BA3 in the right hemisphere was a robust predictor of experienced pain and hypnotizability did not play a significant role in this relationship. During hypnotic hypoalgesia we found that current density at BA3 contralateral to the stimulated hand was a weakly significant predictor (p = 0.046) of perceived pain, while this relation was found to be more robust during hyperalgesia treatment wherein the inclusion of hypnotizability level was highly significant in this relation. Regression analyses using distress rating as a dependent variable were carried out for the hypnotic hypoalgesia alone, since we failed to find significant correlations for control and hyperalgesia treatments in hypnosis (Table 2). This analysis demonstrated a significant (p = 0.031) correlation between distress felt and the activation level at BA3 in the right hemisphere and the effect of adding hypnotizability in this analysis was negligible. In sum, these analyses in the whole showed that current density at BA3 was the only brain response yielding a stable coding for the pain felt across treatments and conditions.

To test in a more direct way the relation between pain modulation and cortical activity, we performed further multivariate regression analyses for each treatment and condition using difference scores of current density as predictors of numerical pain difference scores (see method section). Since in previous analyses we found that current density at BA3 was a stable predictor of the pain felt across treatments and condition, only pain rating and AUC of current density responses at BA3 were used for further regression analyses. Results are presented in Table 5.

**Table 5:** Multivariate regression analysis of changes in pain-related BA3 activation of hypoalgesia and hyperalgesia vs control condition in the left and right hemisphere (LH and RH) in relation to the changes in pain ratings of treatments vs control (Step-1), and after entering SHCS scores as a covariate (step-2).

Waking					Hypnosis			
BA Region	Estimate	SE	t	P	Estimate	SE	t	P
**Control vs Hypoalgesia** ^1^					**Control vs Hypoalgesia** ^7^			
**Intercept**	0.976	0.164	5.97	<0.0001	0.36	0.312	1.15	0.264
**BA3-LH**	0.047	0.017	2.76	0.013	0.407	0.505	0.81	0.427
**BA3-RH**	0.046	0.056	0.82	0.425	-0.123	0.14	-0.88	0.392
**Control vs Hypoalgesia** ^**2**^					**Control vs Hypoalgesia** ^8^			
**Intercept**	0.301	0.302	1	0.334	0.029	0.518	0.06	0.957
**SHCS**	0.267	0.106	2.53	0.022	0.15	0.187	0.81	0.432
**BA3-LH**	0.049	0.049	0.99	0.338	0.347	0.17	2.04	0.06
**BA3-RH**	0.052	0.049	1.07	0.3	-0.079	0.151	-0.52	0.608
**Hyperalgesia vs Control** ^**3**^					**Hyperalgesia vs Control** ^9^			
**Intercept**	-0.067	0.123	-0.55	0.589	0.421	0.3	1.4	0.178
**BA3-LH**	0.126	0.04	3.11	0.006	0.006	0.273	0.02	0.981
**BA3-RH**	0.036	0.036	0.99	0.335	0.328	0.279	1.17	0.257
**Hyperalgesia vs Control** ^4^					**Hyperalgesia vs Control** ^10^			
**Intercept**	-0.786	0.211	-3.73	0.002	-0.876	0.406	-2.15	0.047
**SHCS**	0.291	0.077	3.79	0.002	0.557	0.146	3.82	0.001
**BA3-LH**	0.111	0.03	3.63	0.002	0.046	0.204	0.23	0.823
**BA3-RH**	-0.001	0.029	-0.02	0.986	0.234	0.21	1.12	0.281
**Hyperalgesia vs Hypoalgesia** ^5^					**Hyperalgesia vs Hypoalgesia** ^11^			
**Intercept**	0.816	0.219	3.73	0.002	0	0.572	0	0.999
**BA3-LH**	0.128	0.06	2.13	0.049	0.737	0.196	3.75	0.002
**BA3-RH**	0.097	0.05	1.93	0.07	-0.271	0.192	-1.41	0.176
**Hyperalgesia vs Hypoalgesia** ^6^					**Hyperalgesia vs Hypoalgesia** ^12^			
**Intercept**	-0.37	0.337	-1.1	0.288	-1.008	0.675	-1.49	0.155
**SHCS**	0.493	0.123	4	0.001	0.564	0.246	2.29	0.036
**BA3-LH**	0.095	0.037	2.59	0.019	0.548	0.194	2.82	0.012
**BA3-RH**	0.073	0.046	1.57	0.137	-0.167	0.178	-0.94	0.361

^1^ Step-1: F(2,19) = 2.22, p = 0.139; R-Square = 0.21, Adj. R-Square = 0.11

^2^ Step-2 entering SHCS: F(3,19) = 4.08, p = 0.024; R-Square = 0.43, Adj. R-Square = 0.33

^3^ Step-1: F(2,19) = 9.40, p = 0.0018; R-Square = 0.52, Adj. R-Square = 0.47

^4^ Step-2 entering SHCS: F(3,19) = 16.00, p<.0001; R-Square = 0.75, Adj. R-Square = 0.70

^5^ Step-1: F(2,19) = 7.90, p = 0.0037; R-Square = 0.48, Adj. R-Square = 0.42

^6^ Step-2 entering SHCS: F(3,19) = 15.26, p<.0001; R-Square = 0.74, Adj. R-Square = 0.69

^7^ Step-1: F(2,19) = 9.67 p = 0.0016; R-Square = 0.53, Adj. R-Square = 0.48

^8^ Step-2 entering SHSC: F(3,19) = 6.53, p = 0.043; R-Square = 0.55, Adj. R-Square = 0.46

^9^ Step-1: F(2,19) = 6.89, p = 0.0065; R-Square = 0.45, Adj. R-Square = 0.38

^10^ Step-2 entering SHSC: F(3,19) = 13.13, p =. 0001; R-Square = 0.71, Adj. R-Square = 0.66

^11^ Step-1: F(2,19) = 15.34, p = 0.0002; R-Square = 0.64, Adj. R-Square = 0.60

^12^ Step-2 entering SHSC: F(3,19) = 14.52, p<.0001; R-Square = 0.73, Adj. R-Square = 0.68

A general finding of these regression analyses performed for a waking condition was that, for each treatment, the individual change in current density at BA3 contralateral to the stimulation side, was a stable predictor of the change in pain rating, suggesting a stable pattern of pain coding changes in the brain across all contrasted treatments in waking condition (see left side of Table 5). In addition analyses (step-2) disclosed that hypnotizability level was a significant mediator of these relationships. However, as can be seen in Table 5, the effect of this mediation for hyperalgesia vs control and hyperalgesia vs hypoalgesia differences was that entering hypnotizability in the model enhanced the significance level of pain prediction, while for control vs hypoalgesia differences the effect of entering hypnotizability was to reduce the strength of the relationship from a significant level to a no significant one (Table 5).

Similar analyses performed for hypnosis condition did not yield a significant association between changes in current density at BA3 and changes in pain sensation for Control vs hypoalgesia, and hyperalgesia vs control difference scores (see right side of Table 5), with the exception of hyperalgesia vs hypoalgesia contrast in which change in current density at BA3 in the left hemisphere was a significant predictor of the changes in pain rating. This analysis also showed that effect of entering hypnotizability in the model was to reduce the significance level of this relationship.

### LORETA source localizations of N140 and P200, treatments, conditions and hypnotizability

**Fig 8:**
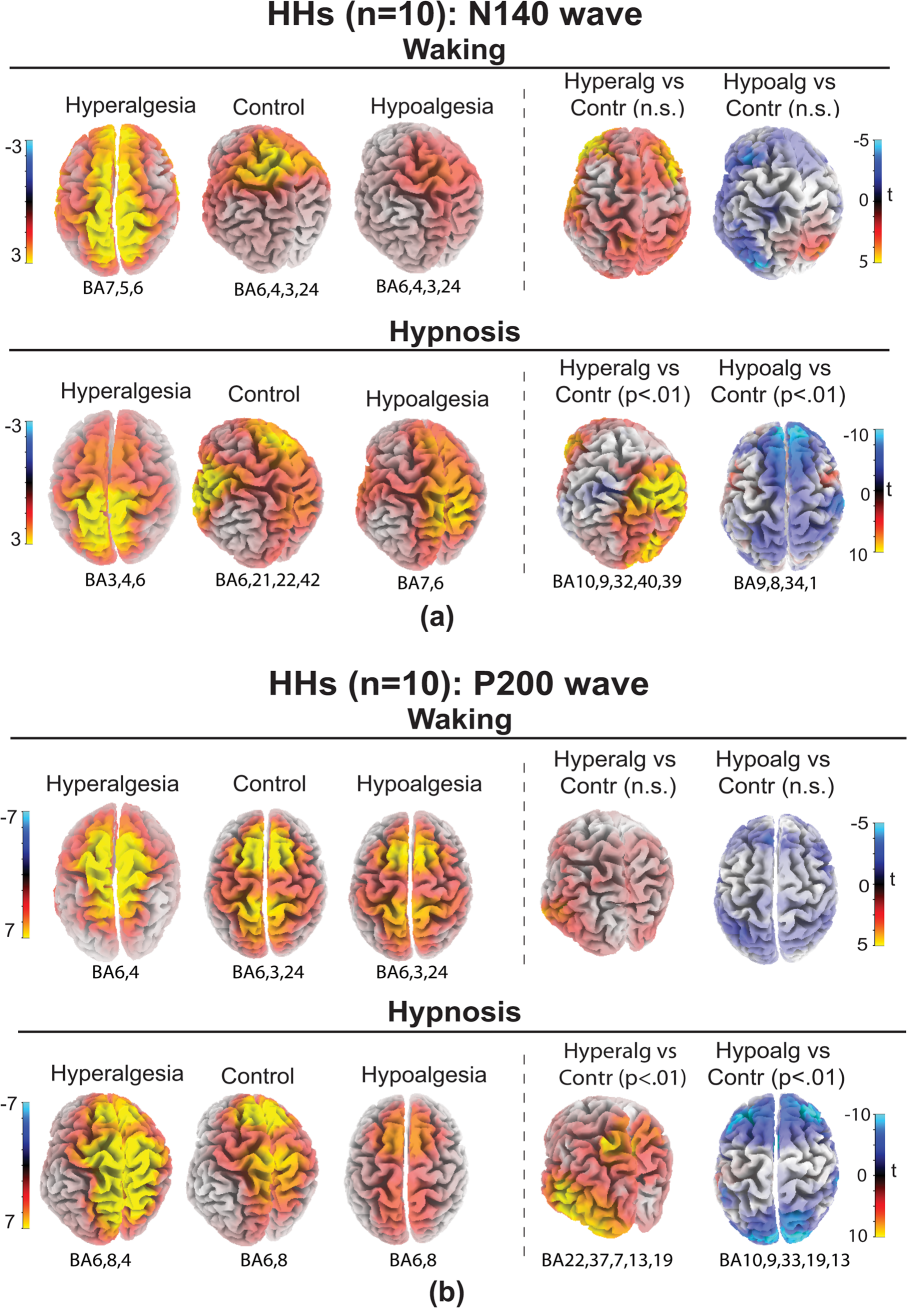
sLORETA solutions modeling the distributed sources for the N140 wave (panel a) and for the P200 wave (panel b) in high hypnotizable participants (HHs) in waking and hypnosis conditions to Hyperalgesia, Control, and Hypoalgesia treatments. Brodmann areas (BA) of estimated sorces (local maxima in yellow color) are reported under each brain map. HHs for Hyperalgesia compared to Control in hypnosis had increased activity (yellow) of N140 wave within middle and superior frontal gyri, anterior cingulate gyrus, and supramarginal gyrus (BA9,10,32,40,39; t-test map, right panel a), and increased activity of P200 wave in the superior (BA22), middle (BA37), inferior and superior temporal (BA19,13) gyri, and superior parietal lobule (BA7; t-test map, right panel b). HHs for Hypoalgesia showed reduced activity (blue) of N140 wave within medial and superior frontal gyri (BA9,8) paraippocampal gyrus (BA34) and postcentral gyrus (BA1; t-test map, right panel a), and reduced activity of P200 wave within middle and superior frontal gyri (BA9 and BA10), anterior cingulate (BA33), cuneus (BA19) and sub-lobar insula (BA13; t-test map, right panel b).

**Fig 9:**
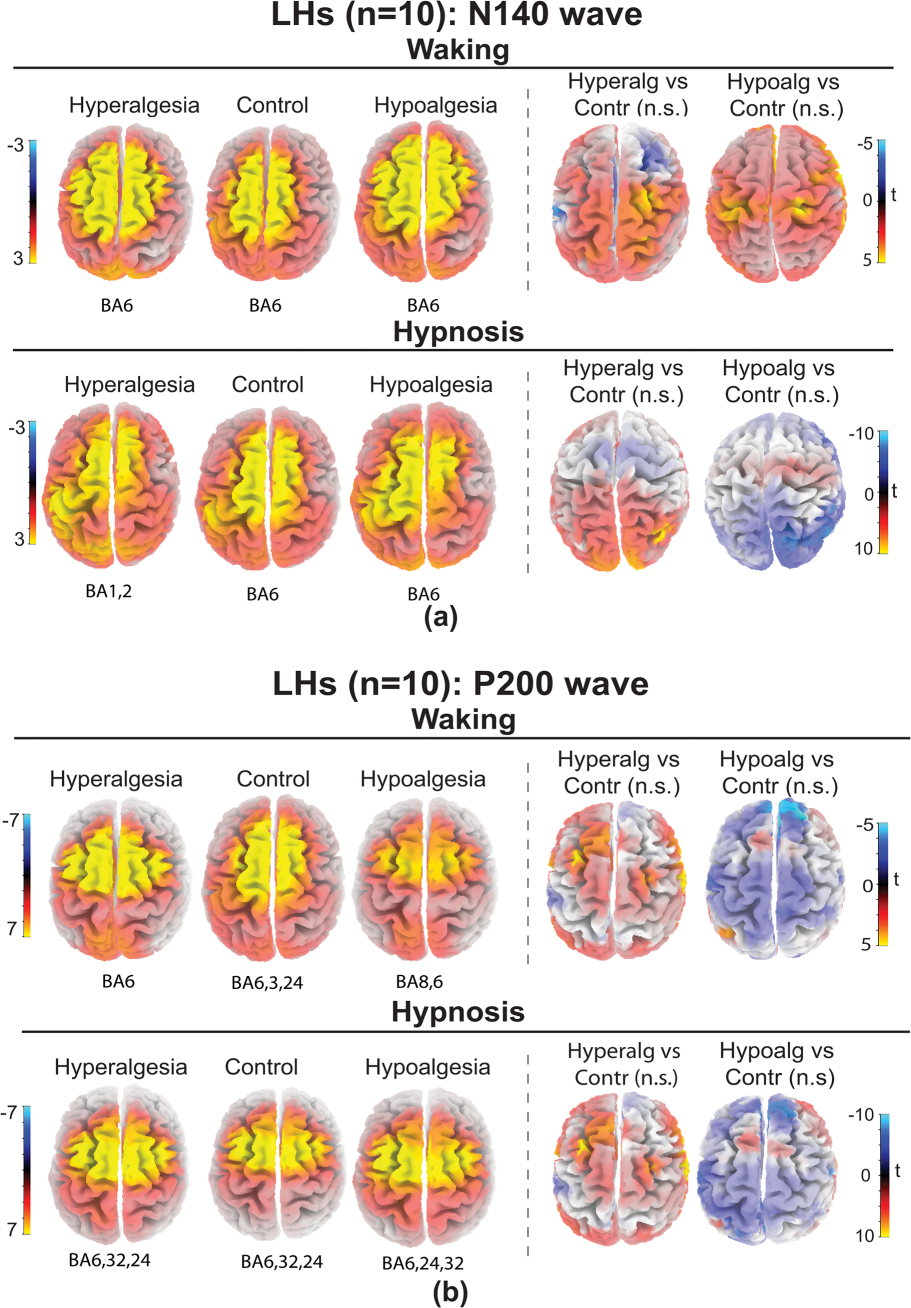
sLORETA solutions modeling the distributed sources for the N140 wave (panel a) and for the P200 wave (panel b) in low hypnotizable participants (LHs) in waking and hypnosis conditions to Hyperalgesia, Control, and Hypoalgesia treatments. Brodmann areas (BA) of estimated sorces (local maxima in yellow color) are reported under each brain map. T-tests for both N140 (panel a) and P200 (panel b) waves between Hyperalgesia vs Control and Hypoalgesia vs Control did not yield significant differences in both waking and hypnosis conditions.

sLORETA source localizations of N140 wave (135–145 ms) obtained for each treatments and t-test maps for HHs are reported in Fig 8A and for LH in Fig 9A. Talairach coordinates and t values are reported in Table 6. T-test analyses performed within a 135–145 ms time window disclosed that in HHs, during hypnosis, hyperalgesia, compared to Control treatment, produced significantly increases of bilateral current density in medial and superior frontal gyri (BA9,10), anterior cingulate gyrus (BA32), middle temporal and supramarginal gyrus (BA39,40). In addition, Hypoalgesia, compared to Control treatment, significantly reduced activity on medial and superior frontal gyri (BA9,8), paraippocampal gyrus (BA34) and postcentral gyrus (BA1). No significant differences in cortical activation between treatments were found within a 135–145 ms time window for LHs both in waking and hypnosis (Fig 9A).

**Table 6:** Brodmann areas (BA) and Talairach coordinates (x, y, z) of statistically stronger cerebral activation for N140 wave in high and low hypnotizable subjects (HHs and LHs) during Hyperalgesia and Hypoalgesia compared to Control treatment.

**HHs during Hypnosis: Hyperalgesia vs Control^1^**						
x	y	z	t*	BA	Lobe	Structure
-5	60	25	6.92	10	Frontal	Superior Frontal Gyrus
5	40	30	13.53	9	Frontal	Middle Frontal Gyrus
5	40	16	7.5	32	Limbic	Anterior Cingulate
54	-57	30	6.86	40	Parietal	Supramarginal Gyrus
54	-62	31	7.25	39	Parietal	Supramarginal Gyrus
**HHs during Hypnosis: Hypoalgesia vs Control** ^2^	** **	** **	** **	** **	** **	** **
-5	50	34	-9.16	9	Frontal	Middle Frontal Gyrus
10	51	39	-6.27	8	Frontal	Superior Frontal Gyrus
20	-1	-13	-6.18	34	Limbic	Parahippocampal Gyrus
64	-22	38	-6.02	1	Parietal	Postcentral Gyrus
**HHs during Waking: Hyperalgesia vs Control** ^3^	** **	** **	** **	** **	** **	** **
-25	57	2	6.92	10	Frontal	Superior Frontal Gyrus
24	47	43	4.61	9	Frontal	Middle Frontal Gyrus
**HHs during Waking: Hypoalgesia vs Control** ^4^	** **	** **	** **	** **	** **	** **
-24	47	36	-3.56	9	Frontal	Superior Frontal Gyrus
25	-75	45	3.92	7	Parietal	Precuneus
-35	-86	23	-4.04	19	Occipital	Superior Occipital Gyrus
**LHs during Hypnosis: Hyperalgesia vs Control** ^5^	** **	** **	** **	** **	** **	** **
20	-93	-12	3.74	18	Occipital	Fusiform Gyrus
-20	92	0	3.08	17	Occipital	Fusiform Gyrus
64	-6	-17	3.96	21	Temporal	Inferior Temporal Gyrus
**LHs during Hypnosis: Hypoalgesia vs Control** ^6^	** **	** **	** **	** **	** **	** **
-25	-61	45	-4.31	7	Parietal	Superior Parietal Lobule
15	-72	27	-4.56	31	Occipital	Precuneus
20	-77	18	-4.33	18	Occipital	Cuneus
**LHs during Waking: Hyperalgesia vs Control** ^7^	** **	** **	** **	** **	** **	** **
14	-19	74	7.44	6	Frontal	Precentral Gyrus
24	-72	52	6.94	7	Parietal	Superior Parietal Lobule
**LHs during Waking: Hypoalgesia vs Control** ^8^	** **	** **	** **	** **	** **	** **
10	-46	48	3.61	7	Parietal	Inferior Parietal Lobule
-15	-76	36	-5.62	19	Occipital	Cuneus

^1^ tcrit = 6.23, p<0.01

^2^ tcrit = 5.03, p<0.01

^3^ tcrit = 5.63, p<0.05

^4^ tcrit = 5.66, p<0.05

^5^ tcrit = 10.51, p<0.05

^6^ tcrit = 10.96, p<0.05

^7^ tcrit = 14.04, p<0.05

^8^ tcrit = 14.80, p<0.05

sLORETA source localizations of P200 wave (210–230 ms) obtained for each treatments and t-test maps for HHs are reported in Fig 8B and for LH in Fig 9B. Talairach coordinates and t values are reported in Table 7. T-test analyses performed for the P200 wave showed that, in HHs during hypnosis, hyperalgesia, compared to control treatment, increased current density at superior (BA22,13), middle (BA37), inferior temporal/middle occipital (BA19) gyri, and superior parietal lobule/precuneus (BA7), while hypoalgesia treatment reduced electrocortical activity at middle (BA9) and superior frontal (BA10) gyri, anterior cingulate (BA33), cuneus (BA19) and sub-lobar insula (BA13). Both for waking and hypnosis in LHs all comparisons, within a 210–230 ms time window, between treatments yielded t-test values under the significance level (Fig 9B).

**Table 7:** Brodmann areas (BA) and Talairach coordinates (x, y, z) of statistically stronger cerebral activation for P200 wave in high and low hypnotizable subjects (HHs and LHs) during Hyperalgesia and Hypoalgesia compared to Control treatment.

HHs during Hypnosis: Hyperalgesia vs. Control^1^						
x	y	z	t*	BA	Lobe	Structure
-54	-48	12	9.74784	22	Temporal	Superior Temporal Gyrus
-54	-63	-1	9.53931	37	Temporal	Middle Temporal Gyrus
-50	-43	21	9.46606	13	Temporal	Superior Temporal
-50	-63	-1	9.29273	19	Temporal	Inferior Temporal/Middle Occipital Gyrus
-30	-55	63	8.98662	7	Parietal	Superior Parietal Lobule/Precuneus
**HHs during Hypnosis: Hypoalgesia vs. Control** ^**2**^						
40	45	25	-8.6906	10	Frontal	Middle Frontal Gyrus
40	40	30	-8.79829	9	Frontal	Superior Frontal Gyrus
-5	20	17	-9.29295	33	Limbic	Anterior Cingulate
30	15	13	-8.89571	13	Sub-lobar	Insula
25	-86	32	-9.1781	19	Occipital	Cuneus
**HHs during Waking: Hyperalgesia vs. Control** ^**3**^						
-53	-40	-18	3.17	37	Temporal	Inferior Temporal Gyrus
**HHs during Waking: Hypoalgesia vs. Control** ^**4**^						
54	4	-21	-4.45	21	Temporal	Middle Temporal Gyrus
**LHs during Hypnosis: Hyperalgesia vs. Control** ^**5**^						
-53	16	37	5.33	9	Frontal	Middle Frontal Gyrus
-53	-22	-24	5.17	20	Temporal	Inferior Temporal Gyrus
**LHs during Hypnosis: Hypoalgesia vs. Control** ^**6**^						
30	32	40	-5.72	9	Frontal	Middle Frontal Gyrus
-55	-54	40	-4.89	40	Parietal	Inferior Parietal Lobule
**LHs during Waking: Hyperalgesia vs. Control** ^7^						
-35	46	42	5.19	9	Frontal	Middle Frontal Gyrus
-60	-53	-15	5.04	37	Temporal	Inferior Temporal Gyrus
**LHs during Waking: Hypoalgesia vs. Control** ^8^						
49	47	4	-3.89	10	Frontal	Middle Frontal Gyrus
-40	-31	52	-4.12	40	Parietal	Postcentral Gyrus
50	-32	52	-3.64	40	Parietal	Postcentral Gyrus

^1^ tcrit = 8.35, p<0.01

^2^ tcrit = 8.28, p<0.01

^3^ t crit = 4.61, p<0.05

^4^ t crit = 5.43, p<0.05

^5^ t crit = 13.56, p<0.05

^6^ t crit = 14.21, p<0.01

^7^ t crit = 14.04, p<0.05

^8^ t crit = 14.80, p<0.05

## Discussion

In this paper we describe a SERP study examining the effects of hypnotic susceptibility and hypnotic suggestions on electro-cortical responses and on sensory-discriminative (pain rating) and affective-motivational (distress rating) components of pain induced by noxious electric stimulation. These measures were obtained in waking and hypnosis condition under a relaxation-control and suggestions to either increase (hyperalgesia) or decrease pain sensation (hypoalgesia). In this context, we did an attempt to validate previous pain-ERP findings to noxious electric stimulation during hypnosis [49]. We asked three main questions: 1) do high and low susceptible individuals respond differentially to the experience of pain; 2) do hypnotic suggestions influence the experience of pain; and 3) are there physiological mechanisms that differentially mediate the manner in which high and low susceptible individuals respond to these suggestions. Using sLORETA tool, the present study served to highlight brain responses and cortical regions coding for pain/distress and to evaluate if the brain pattern of pain/distress coding may change depending on the experimental treatment and/or condition. The main aim of the study was to evaluate how treatments of hypoalgesia and hyperalgesia, as compared to a relaxation-control, differentially affected subjective pain ratings and somatosensory event-related potentials (SERPs) to noxious electric stimuli in waking and hypnosis and how these differences are reflected in HH and LH participants. Source localization analysis (sLORETA method) of N100 and P200 SERP waves was used to substantiate the role of the main cortical regions sensitive to pain modulation treatments in HH and LH participants during waking and hypnosis.

### Pain and distress

We found little differentiation between pain and distress scores across experimental treatments, indicating that suggestions for pain control influenced in a similar fashion both perceptual and affective expressions of pain. LH group showed little differentiation between hypoalgesia and hyperalgesia treatments in waking and hypnosis conditions, with the exception that they disclosed significant pain and distress reduction during hypoalgesia and hyperalgesia compared to control treatment in waking condition. In contrast, HHs showed more pronounced rating changes in the instructed direction, an effect that was amplified during hypnosis (Fig 1). We think that the reduction in pain and distress during hyperalgesia, as compared to control treatment during waking, in LH subjects, may be due to the nature of hyperalgesia treatment itself and to the reduced cognitive capacity of these subjects, as compared to HHs, to focus attention on the stimulated finger and to form a visual image devoted to increase pain sensation [50–53]. This task, in LHs, becomes more difficult in waking condition in which the capacity to focus attention on task-requirement is reduced as compared to hypnosis. In addition, we cannot exclude that the reduction of pain perception during waking hyperalgesia treatment in these subjects may be due to an effect of enhanced stimulus predictability, which reduces pain in the healthy population and/or habituation [89,90]. Hyperalgesia suggestions in HHs during hypnosis, wherein the effect of suggestion is amplified [50], may have contrasted the mechanisms reducing pain as stimulus predictability and/or the mechanisms sustaining habituation. These results are in line with previous reported findings [49,50,57,91–95].

### N140 and P200 waveforms

Scalp distribution of N140 amplitude showed a pronounced lateralization to the left hemisphere, contralateral to the side of noxious stimulation (Fig 4). This finding clearly indicates that this SERP component is stimulus oriented [49,58,96]. Interestingly, during hypnosis, HHs had significant amplitude decreases of both N140 and P200 waves induced by hypoalgesia treatment, and amplitude increases by hyperalgesia treatment, as compared to a control treatment (for N140 see top panel of Fig 2, and for P200 see top panel of Fig 5). For the N140 wave main changes were observed across left-frontal and frontocentral sites (top-right panel of Fig 4), for the P200 wave over the left-frontocentral, central, and bilateral centroparietal and parietal sites (top right-panel of Fig 6). These treatment-induced differences disappeared in waking condition (top left-panel of Fig 6), while LH subjects did not disclose significant SERP amplitude changes across treatments for both waking and hypnosis conditions (see bottom panel of Fig 6). In terms of N140 wave, our findings in HHs, contradict previous statements that this component, being stimulus oriented, cannot be modulated by specific hypnotic suggestions [49,52]. Although there is experimental evidence that the somatosensory N140 wave can be modulated by selective attention [97–99], the direction of changes of both pain experience and N140 amplitude were convincingly indicating effects associated to the nature of hypnotic suggestions rather than to nonspecific effects of attention.

Research has demonstrated that both N140 and P200 waves are enhanced in amplitude to stimuli coming by the attended hand and reduced to stimuli coming from the unattended hand [39, 97, 99], and reduced in HHs by hypnotic obstructive hallucination [48]. Since in the present study hypoalgesia and hyperalgesia treatments did not differ in terms of attentional set (i.e., both treatments required subjects to focus attention on the stimulated hand and to produce mental images devoted respectively to reduce and enhance pain sensation), we exclude that differences in an unspecific effect of focused attention alone between treatments may account for the observed N140 amplitude changes between treatments. Rather we are brought to assume that, for hypoalgesia treatment, both the obstructive nature of mental image itself, and its improved representation in HHs during hypnosis, could have produced the smaller amplitudes of N140 and P200 waves as compared to a control treatment. The same can be said for hyperalgesia treatment during hypnosis in HHs, i.e., that the suggested mental image of a vise that gripped the finger as the stimulation time progressed, in parallel with pain sensation, could have enhanced stimulus saliency [39]. Thus, we cannot exclude that, during hyperalgesia treatment, saliency neural detectors, as predicted by Legrain’s model [1], may have contributed to the frontocentral P200 modulation in HHs, which in turn would be a measure of orienting to the most significant alerting condition. Furthermore, we think that the lacking differences in LHs for these SERP waves, observed among treatments and conditions, both in waking and hypnosis, may be due mainly to the reduced capacity of these subjects to focus attention necessary to form the mental images designed to reduce pain sensation during hypoalgesia and for pain amplification during hyperalgesia treatment. This may have prevented the HHs, during hyperalgesia treatment, to fulfill task requirements necessary to contrast the mechanisms reducing pain as stimulus predictability and/or the mechanisms sustaining habituation [89,90]. This interpretation is supported by research findings showing that LHs usually possess weaker abilities to focus and sustain their attention as well as to ignore irrelevant stimuli than do HHs and these differences are reflected in underlying brain dynamics [100].

In addition, since the P2 wave is believed to reflect the painfulness of the stimuli [74,94], we think that both P200 and N140 amplitude changes associated to hypoalgesia or hyperalgesia effects during hypnosis, may reflect, respectively, the operation of a top-down inhibition or sensitization process of pain sensation [49,57,101,102]. This conclusion seems in agreement with Horton and colleagues neuroimaging findings of a larger rostrum in HHs, compared to LHs, indicating a more efficient mechanism of sensory gating in the former than the latter [53,103].

However, it must be stressed that the obtained findings are limited by the fact that ERPs of the present study were elicited using transcutaneous electric stimulation. Even if the stimuli produced a percept qualified as painful, it can be expected that the elicited ERPs were mainly related to the activation of non-nociceptive A-β afferents and the lemniscal pathway rather than to the activation of A-δ and C fibers [104]. This crucial point may explain the little differentiation we observed between perceptual and affective expressions of pain.

In line with general models of selective attention [105] and their application in hypnosis [106] (Egner et al., 2005), the observation that hyperalgesia and hypoalgesia suggestions modulate the magnitude of the N140 and P200 waves (Figs 3, 4 and 6) may be seen as the product of the interplay of excitatory and inhibitory fronto-centroparietal processes [107] during monitoring and orienting of attention toward noxious events [108]. Thus, we cannot exclude that the increased N140 and P200 waves in HHs, during hyperaglesia suggestion in hypnosis, reflect neural processes involved in involuntary shifts of attention toward the expected unpleasant sensory event. We think, however, that this modulation process may be of a global nature since it involves not only the P200 wave, but also the N140 wave which is believed to represent mainly an index of ‘bottom-up’ processing of the ascending nociceptive input [58]. A similar inhibitory effect, involving negative and positive SERP components, has been also reported in previous hypnosis studies using obstructive imagery of incoming somatosensory stimuli [48–51]. Unfortunately, due to the short ISI of 0.86 ms (chosen to compare and extend the findings reported in a previous hypnosis-pain study [49]), we did not collect pain and distress ratings after each noxious stimulus. This approach may have had severely introduced behavioral and neural habituation/sensitization patterns which prevents comparison with previous studies using ratings from single stimuli and long ISIs (e.g., 7–14 sec; [34,35,52]). However, this aspect of the design may open an entirely new perspective on the study, leaving room for an original question on the effect of hypnosis on pain-related habituation/sensitization.

### Investigation of pain coding across experimental conditions using sLORETA

sLORETA source analysis of ERP response to noxious electric stimulation revealed clusters of significant activation as ROIs, including the BA3, BA6, and BA24 cortical regions in the left hemisphere contralateral to the stimulated side. These clusters of significant activations were temporally localized within a time window of 190–270 ms, clearly within the latency variability range of the P200 wave. In terms of ROIs, these findings parallel previous reports of the most commonly activated cortical pain-related regions reported in imaging studies, namely the primary somatosensory cortex, the frontal lobe, and anterior cingulate cortex [109–114]. Interestingly, activity in each contralateral ROI was found positively correlated with both subjective pain ratings in a waking-control condition (Table 2). However, the most robust association was found between current density at BA3, contralateral to the stimulated hand, and pain level since this relation was stable across treaments both in waking and hypnosis conditions (Table 2). In contrast, the relation of activity in BA3 with distress level was less stable across conditions, but a significant correlation was found between activity in the left-sided BA24 and distress level, indicating, in line with previous fMRI findings [56, 115,116], the involvement of anterior cingulate in coding the affective-motivational dimension of pain (Table 2). The present results obtained in waking condition parallels and extend previous heat-pain findings [17] to electric-pain stimulation. As is well known, pain is a complex experience involving at least two dimensions: sensory-discriminative dimension and affective-motivational dimension [117], these findings suggest that pain, and to a less extent distress level, significantly co-varies with the late activity in the primary somatosensory cortex within a time window including the P200 SERP component. This issue is very relevant, as some of the above mentioned studies, using longer ISIs, already illustrated the lack of co-variation between the early somatosensory activity and the intensity of pain sensation (e.g., [34,35]). More, our findings reaffirm the important roles of primary somatosensory, midfrontal and cingulate cortices in pain perception. The prefrontal and cingulate cortices are believed to be mainly associated with affective-motivational aspects of pain [118,119]. Several past PET and fMRI studies showed that the activation of ACC and medial prefrontal cortex correlates with affective reports of pain [56, 115,116]. Slightly different from the existing evidence, in the present study the correlations obtained between activities in the primary somatosensory cortex and pain sensation in waking-control condition were more robust than that observed for distress rating. This discrepancy is likely due to the fact that for the participant may be easier to rate pain rather than distress sensation, or to distinguish between different aspects of pain sensation [117,120].

Our multiple regression findings, testing the association between subjective pain/distress ratings and activity in the ROIs, disclosed that current density measure at BA3, contralateral to the stimulated side, was the only brain response yielding a stable coding for the pain felt across treatments in both waking and hypnosis conditions (Tables 3 and 4). These findings were further confirmed by multivariate regressions, testing more directly, how changes in current density from each treatment (vs control) were related to changes in pain ratings (vs control). In the whole these findings suggest that changes in brain activity at BA3 reflects a stable pattern of pain coding changes across all contrasted treatments in waking condition (Table 5). These last analyses also disclosed that the effect of hypnotizability was to enhance the significance level of the relationships between pain modulation and brain activity for changes of Hyperalgesia vs Control and Hyperalgesia vs Hypoalgesia, while for changes of Control vs Hypoalgesia, the effect of hypnotizability was to reduce the relationship from a significant level to a no significant one (see Table 5). Importantly, regression analyses showed that hypnosis reduced the strength of the association of pain modulation and brain activity change at BA3 (Table 5), with the exception of hyperalgesia vs hypoalgesia contrast in which change in current density at left sided BA3 was significantly associated with changes in pain rating. The finding that hypnosis reduces the strength of the association between pain sensation and brain activity is in line with original findings that dissociation in hypnosis cancels the relationship between EEG gamma (32–100 Hz) and perceived pain observed in waking condition [121,122].

### Sources of N140 and P200 waves

Two contrasting conceptual positions have emerged in the literature of pain research. One position stated that functional imaging data may contain a genuine and objective signature of the painful experience [123, 124] so that activation of the anterior insulae and cingulate has sometimes been equated with physical pain, leading to questionable conclusions such as that “social rejection hurts physically” (e.g., [125,126]). The other position stated that the neocortex does not have any tissue specifically dedicated to pain and suggests that PM represents a nonspecific salience-detection system for the body, activated by relevant events regardless of the sensory channel through which these events are conveyed [120,124]; review [127]. Our N140 and P200 source localization findings appear to support a view of PM, in between these extreme positions, as conceptualized by a few investigators (e.g., [24,25,113]) and recently reconsidered [11] that the pain matrix cannot be unequivocally defined, the role of different regions being dependent on the context in which the stimuli are delivered. According to this view, pain and distress modulations result from continuous interaction of these pain matrices, and substantial changes in the pain experience can be obtained by acting on each of them. On this basis, we attempted to discuss our source localization findings.

Our sLoreta analysis of N140 wave showed that, during hypnosis, hypoalgesia, compared to a control treatment, had significantly reduced activity in both hemispheres within postcentral gyrus (BA1), medial and superior frontal gyri (BA9,8), and paraippocampal gyrus (BA34; see Fig 8 and Table 6).

According to the new conceptualization of pain matrix [11], BA1 (part of the primary somatosensory cortex S1), is considered the main sensory receptive area for the sense of touch, which is highly aligned with nociceptive maps of painful stimuli [18,20,128], but its activity is possibly driven by innocuous A-β afferents and the lemniscal fibers induced by the electrical stimuli. Thus, inferences on S1 activity and modulation being related to a first-order nociceptive matrix should be ruled out. However, this region appears as a necessary entry to generate physiological pain experiences and the frontal regions of BA9 and BA8, which are considered part of the classical second-order pain matrix, are necessary for the transition from cortical sensation to conscious pain and its multiple attentional-cognitive modulations [11,58,129]. Further, BA34 is located in the parahippocampal gyrus at temporal pole, which is believed part of third-order network which considered to account for changes in the emotional component of pain experience as anxiety and fear [34,130–133]. Notably, it has been shown that pain-relieving effects derived from placebo [134,135], relief context [136], and strong religious beliefs [137] or self-control over the stimulus [138,139], are associated with activity changes in the third-order pain matrix.

Our sLORETA findings for Hyperalgesia treatment in hypnosis disclosed a significant amplitude increase of bilateral N140 wave activity in medial and superior frontal gyri (BA9,10), anterior cingulate (BA32), middle temporal and supramarginal gyrus (BA39,40). These findings are also in line with PM notion suggesting these cortical regions as part of the conceptualized second-order and third order pain matrix network [11].

In terms of the P200 wave we found during hypnosis that in HH participants the hypoalgesia treatment produced a reduced activity at middle (BA9) and superior frontal (BA10) gyri, anterior cingulate (BA33), cuneus (BA19) and sub-lobar insula (BA13). In contrast, hyperalgesia treatment produced an increased activity at superior temporal (BA22,13), medium temporal (BA37), inferior temporal/middle occipital gyri (BA19) and at superior parietal lobule/precuneus (BA7; Fig 8 and Table 7). These observations parallel findings showing that, for pain modulation, the insula is constantly activated within the pain matrix [14,140] to underpin the transformation of sensory events into vegetative reactions and associated internal feelings [141–143].

In the whole, the above mentioned findings suggest that reduced and enhanced activity in these higher-order contextual matrices may influence nociception via top-down projections by changing the sensory gain at the source, which is, in cortical receiving areas [144], thalamus [145], and even at the brainstem [146]. This conclusion is in line with previous findings showing that, depending on hypnotic suggestions, the activation of the second and third order matrices can be necessary for pain modulation in hypnosis [56,89,147]. Moreover, the finding that the HH group was the most sensitive to pain modulation treatments endorses the hypothesis that mechanisms of sensorimotor gating are more efficient in HHs [53,105] and suggests that the cognitive section of the anterior cingulate plays a role in pain modulation, which together with prefrontal and posterior parietal areas, sustain attention and evaluative processes of cognitive control [148].

Our most striking source activation findings fit well with the suggested cortical regions outlined in the re-conceptualization of pain matrix [11] and suggest that hypnotic suggestions of hypoalgesia or hyperalgesia are respectively associated with a dramatically reduction and enhancement of cortical activity within the second- and third-order pain matrices (Figs 8 and 9). These findings extend the current knowledge on hypnotic modulation of brain activity in a nonclinical sample [47–49,52,56,149]. However, it is important to point out that current source findings are purely speculative and must be considered with caution, since they were obtained using only 30 scalp electrodes and the modeling relied on a standard head model (instead of individual MRI data). With an impaired spatial resolution, there is a smaller chance that sLORETA will be able to separate two closely spaced sources [150,151]. More studies are necessary to replicate our findings using an enhanced spatial resolution. Another limitation of the present study is that our findings are restricted to women participants and, thus, cannot be generalized to men. Further studies would do well to consider gender, time of day, eye-recording asymmetry factors, and heterogeneity of hypnotizability as potential mediators of pain responses.

## Conclusions

The present findings describe hypnotic modulation of brain activation patterns associated with nociceptive processing. Correlation analyses distinguished BA3 activity, contralateral to the stimulation side, as the only one reflecting a stable pattern of pain coding changes across treatments in waking and hypnosis conditions. A more direct regression analysis testing also showed that hypnosis reduced the strength of the association of pain modulation and brain activity changes at BA3. The study convincingly demonstrates that hypnotic hypoalgesia is associated with reduced activity of the N140 and P200 SERP components, whereas hypnotic hyperalgesia is associated with increased activity of these components. Our source findings are among the first to clearly distinguish separate regions of the first, second, and third order pain matrices [11] as sensitive to hypoalgesia and hyperalgesia treatments during hypnosis. We suggest that treatments for reducing and increasing pain sensation were effective in pain modulation through top-down influences.
